# Skeletal muscle differentiation of human iPSCs meets bioengineering strategies: perspectives and challenges

**DOI:** 10.1038/s41536-022-00216-9

**Published:** 2022-04-07

**Authors:** Federica Iberite, Emanuele Gruppioni, Leonardo Ricotti

**Affiliations:** 1 The BioRobotics Institute, Scuola Superiore Sant’Anna, 56127 Pisa (PI), Italy; 2 Department of Excellence in Robotics & AI, Scuola Superiore Sant’Anna, 56127 Pisa (PI), Italy; 3 Centro Protesi INAIL, Istituto Nazionale per l’Assicurazione contro gli Infortuni sul Lavoro, 40054 Vigorso di Budrio (BO), Italy

**Keywords:** Stem-cell differentiation, Tissue engineering, Stem-cell biotechnology

## Abstract

Although skeletal muscle repairs itself following small injuries, genetic diseases or severe damages may hamper its ability to do so. Induced pluripotent stem cells (iPSCs) can generate myogenic progenitors, but their use in combination with bioengineering strategies to modulate their phenotype has not been sufficiently investigated. This review highlights the potential of this combination aimed at pushing the boundaries of skeletal muscle tissue engineering. First, the overall organization and the key steps in the myogenic process occurring in vivo are described. Second, transgenic and non-transgenic approaches for the myogenic induction of human iPSCs are compared. Third, technologies to provide cells with biophysical stimuli, biomaterial cues, and biofabrication strategies are discussed in terms of recreating a biomimetic environment and thus helping to engineer a myogenic phenotype. The embryonic development process and the pro-myogenic role of the muscle-resident cell populations in co-cultures are also described, highlighting the possible clinical applications of iPSCs in the skeletal muscle tissue engineering field.

## Introduction

Skeletal muscles enable voluntary movements and, consequently, a series of dynamic interactions between individuals and their surrounding environment. In vivo, skeletal muscles can self-regenerate: after traumas or other tissue damage, resident muscle stem cells named muscle satellite cells (MuSCs), are activated. MuSCs are located between the cell membrane and the basal lamina of myofibers, and their activation leads to cell proliferation and eventually exit at G1 phase. MuSCs then fuse to form terminally differentiated multinucleated myofibers, thereby restoring the pool of highly specialized cells in the tissue^1^.

However, the skeletal muscle’s ability to self-repair may be impaired due to aging, genetic diseases^2^, or injuries with volumetric muscle loss (VML)^3^.

In such cases, having healthy patient-specific muscle grafts developed in vitro and ready to be implanted in the impaired area would be highly desirable to restore tissue functionality. Fully-functional muscle grafts could also be exploited in lab-on-a-chip platforms for testing the efficacy, toxicity, and possible side effects of drugs, thereby significantly reducing (ideally eliminating) the need for animal sacrifices^4^.

To obtain such muscle grafts in vitro, appropriate myogenic precursors in a three-dimensional (3D) construct need to be engineered, pushing their differentiation to match the morphological and functional features of the native human muscle tissue. This is thus the objective of skeletal muscle tissue engineering, which aims to harness the knowledge derived from studying embryogenesis processes, and partly reproducing them in vitro.

Induced pluripotent stem cells (iPSCs) were generated for the first time in 2006 by Shinya Yamanaka^5^ and marked a crucial milestone in the field of biomedical sciences. These cells exhibit both transcriptional and epigenetic signatures similar to those of embryonic stem cells (ESCs), and thus they open up exciting scenarios for tissue engineering. Using human iPSCs, it is theoretically possible to create tissues or organs with patient-derived cells, thus eliminating immunogenicity issues. Furthermore, patient-specific tissues/organs-on-a-chip can be created, on which drugs can be tested in a customized way. The most effective and safest drug could be first tested on the custom patient-reflective chip, before administering it to the subject. Consequently, iPSCs also have an enormous potential in the field of skeletal muscle tissue engineering.

Several biochemical protocols for the myogenic induction of iPSCs have been proposed. Some recent reviews analyze and compare the different approaches pursued^6–10^. However, in almost all of them, the focus is only on the biochemical stimuli affecting stem cell fate.

Two main approaches have been described: (1) transgenic approaches by the overexpression of master myogenic transcription factors (e.g., *MYOD1*, *PAX3/7*), and 2) directed differentiation with stepwise induction using small molecules and growth/differentiation factors. These two approaches focus on creating myogenic progenitors in vitro (from both healthy and unhealthy donors), which are often subsequently differentiated into myotubes. For clinical applications of these cells, in vitro differentiation of muscle progenitor terminal is performed to evaluate their myogenicity. This potential is then validated with xenotransplantation and the evaluation of the progenitors’ ability to create new myotubes and repopulate the stem cell niche (see Tables 1 and 2)^11–20^. Being able to repopulate the stem cell niche is key to ensuring long-term homeostasis in future tissue regeneration requirements.

**Table 1:** Transgenic approaches for human induced pluripotent stem cells skeletal muscle differentiation. The bibliographic research was performed until December 2020 using PubMed and SCOPUS databases. The search queries used for titles and/or abstracts starting from 2006 (year of the first report on iPSCs) were [(induced pluripotent stem cells) AND (skeletal muscle)]; [(skeletal muscle cell) AND (differentiation) AND (induced pluripotent stem cells)]; [(induced pluripotent stem cells) AND (myogenesis) OR (myogenic differentiation)]. Review articles, book chapters, and conference abstracts/papers were not included.

Ref.	iPSC origin and lines	Culture conditions in proliferation	Transgene and overexpression system	Myogenic progenitor derivation^a^	Terminal differentiation^a^	Culture type	Myogenic cell selection	Functional tests	In vivo engraftment
Darabi et al., 2012^11^	Fibroblasts (IPRN13.13, IPRN14.57)	Matrigel^®^ coating with mTeSR1	*PAX7* (dox-inducible lentiviral system)	IMDM, 15% FBS, 10% HS, 1% chick embryo extract, 50 µg/mL AsAc, 4.5 mM MTG (11 days); same medium as before with dox 0.75 μg/mL (4 days); sorted for PAX7+ (GFP+); same medium as before with dox 0.75 μg/mL and human FGF2 (5 ng/mL) (2 weeks) **Markers** GE: *T, MYOD1* PE: MRFs, CD29/44, CD56/63/105, CDH15, ITGA7	logDMEM, 5% HS (7 days) **Markers** GE: *DMD, MYH* PE: MYH, MYOG	EBs and then adherent	Progenitor purification (EBs): FACS for iPAX7+ (GFP+)	n/a	**Cell type:** myogenic progenitors iPAX7+ **Endpoint:** 2 months **Outcome:** successful engraftment with fibers human DMD+ and LMNA. Increase in tetanic, absolute, and specific force, but no influence on fatigue tests. No tumor formation after 46 weeks (injected in TA muscles of NSG mice)
Tedesco et al., 2012^12^	Fibroblast and myoblasts	iMEFs with KO DMEM; 25% KOSR, 2 mM L-glut, 1 mM Na pyr, 100 IU mL pen, 100 mg/mL strep, 1% NEAA, 0. 2 mM 2-ME, 10 ng/ml human FGF2	*MYOD1* (tamoxifen-inducible lentiviral system)	Generation of mesangioblast-like cells (HIDEMs): Matrigel^®^ coating with α-MEM, pen (100 IU/mL), strep (100 mg/mL), 10% FBS, 1% NEAA, 0.2% 2-ME (14 days); MegaCell DMEM (7 days) **Markers** GE: n/a PE: CD44, CD13, CD146, PDGFRA, MYOD1	4OH-tamoxifen or standard tamoxifen (5 days) **Markers** GE: *MYOD1, MYOG, SGCA* PE: MYH, MYOD1	Adherent	Progenitor purification: FACS for SSEA1-	n/a	**Cell type:** HIDEMs from LGMD2D iPSCs GFP+ **Endpoint:** 1 month **Outcome:** 5–7% cell survival, SGCA+ fibers are present and DPC was reconstituted (fibers SGCB+/SGCG+ ). Increase in tetanic force ex vivo. Repopulation of alkaline phosphatase+ pericyte pool.(injected in TA muscles of Sgca^null^/scid/beige mice)
Tanaka et al., 2013^13^	Fibroblasts (201B7, 253G1, 253G4)	Collagen I or Matrigel^®^ coating with primate ES medium with 4 ng/mL FGF2	*MYOD1* (dox-inducible PiggyBac transposon-Tet-ON system)	Collagen I or Matrigel^®^ coating; primate ES medium, 10 µM ROCK inhibitor (1 day), added 1 µg/mL dox (1 day); α-MEM, 5% KOSR, 50 mU/L pen/50 mg/L strep, and 100 mM 2-ME (5 days) **Markers** GE: *CKM, MYOD1, MEF2C* PE: n/a	DMEM with 5% HS, 50 mU/L pen, 50 mg/L strep, 10 ng/mL IGF-1, 2 mM L-glut and 100 mM 2-ME (7 days) **Markers** GE: *MYOG, DMD*, microarray PE: DMD, MYH, ACTA1	Adherent	FACS for iMYOD1+ (mCherry+)	Contraction upon ESt at day 14 (100 V, 3 ms, 1 Hz)	**Cell type:** myogenic progenitors (day 6 of differentiation) **Endpoint:** 28 days **Outcome:** cells displayed fusion potential and the expression of human DMD and human SYNE1 (injected in TA muscles of NOD/Scid-DMD mice)
Abujarour et al., 2014^24^	FTc01-C1 and FTc01-C2	Matrigel^®^ coating with DMEM/F12, 20% KOSR, 1% NEAA, 2 mM L-glut, 100 mM 2-ME, 10 ng/mL FGF2	*MYOD1* (dox-inducible lentiviral system)	DMEM, 10% FBS, 1 µg/mL dox (4 days) **Markers** GE: n/a PE: n/a	logDMEM, 5% HS (3 days) **Markers** GE: n/a PE: MYOD1, MYH, MYOG	Adherent	n/a	n/a	n/a
Quattrocelli et al., 2015^14^	Fibroblasts and mesangioblasts	iMEFs with DMEM/F12 with 20% KOSR, 1% pen/ strep, 1% L-glut, 1% NEAA, 0.2% 2-ME, and 5 ng/mL FGF2	*PAX3/7* (transient overexpression by pSPORT6.1 plasmid)	DMEM, 2% HS, 1% ITS, 100 ng/mL noggin, 1 mM TGF-β inhibitor (10 days) **Markers** GE: *T, MEOX1, TBX5, PAX3/7, KDR* PE: MYH2	n/a	Adherent	PDGFRA/B+CD44+ of the EBs before differentiation	n/a	**Cell type:** iPAX3/7 cells PDGFRA/B+CD44+ **Endpoint:** 2 months **Outcome:** muscle fiber regeneration with SGCB expression in Sgcb^null^/Rag2^null^/ γc^null^ mice and DMD expression in dystrophic mouse
Shoji et al., 2015^25^	Fibroblasts	iMEFs with primate ES medium with 4 ng/mL FGF2	*MYOD1* (dox-inducible PiggyBac transposon-Tet-ON system)	Matrigel^®^ or collagen I coating, 20% KOSR replacement media, 100 μ g/mL neomycin sulphate (1 day); 20% KOSR replacement media, 1 μg/mL dox (1 day); 5% KOSR/α-MEM media, dox, 2-ME (5 days) **Markers** GE: *MYOD1, MYOG, DMD, CKM, TPM2* PE: n/a	DMEM, 2% HS (7 days) Markers GE: *DMD, TPM2* PE: ACTA1, CKM, MYH, ultrastructure	Adherent	Myogenic progenitors: FACS for iMYOD1+(mCherry+)	Detection of Ca^2+^ influx upon ESt (12 V, 50 ms, 0.2 Hz) (day 9 of differentiation)	n/a
Lenzi et al., 2016^26^	Fibroblasts	hESC-qualified Matrigel^®^ coating with Nutristem-XF	*MYOD1* (dox-inducible PiggyBac transposon-Tet-ON system)	DMEM/F12 with GlutaMAX^TM^, 20% KOSR, 1X NEAA, 100 U/mL pen, 100 μg/mL strep, 0,1 mM 2-ME, (5 days); 200 ng/mL dox (2 days) **Markers** GE: *T* PE: n/a	Skeletal Muscle Cell Differentiation Medium (Promocell), 100 U/mL pen, 100 μg/mL strep, 200 ng/mL dox (5 days) **Markers** GE: *DMD, MYOD1, MYOG, MYH, MEF2C* PE: MYH2, MYOG	Adherent	n/a	Patch-clump recordings of ACh-evoked currents, and intracellular Ca^2+^ release with ACh stimulations	n/a
Uchimura et al., 2017^51^	414C2, 409B2	Matrigel^®^ coating with StemFit AK02N, 0.5% pen/strep	*MYOD1* (tetracycline-inducible system)	StemFit AK02N, 0.5% pen/strep (1 day); primate ES Cell media, 0.5% pen/strep (1 day); same medium as before, 1 μg/mL dox (1-2 days); α-MEM, 0.5% pen/strep, 5% KSR, 200 μM 2-ME, 1 μg/mL dox (6–7 days) **Markers** GE: *MYOD1, MYOG, MYH* PE: MYH2, MYOG	DMEM, 0.5% pen/strep, 2 mM L-glut, 200 μM 2-ME, 5% HS, 10 ng/mL IGF-1, 5 μM SB431542 (2-3 days); DMEM, 0.5% pen/strep, 2 mM L-glut, 200 μM 2-ME, 2% HS, 10 ng/mL IGF-1, 5 μM SB431542 (7 days) **Markers** GE: n/a PE: MYH2	Adherent	n/a	n/a	n/a
Sato et al., 2016^204^	n/d	iMEFs with DMEM/F12, 20% KOSR, 1% GlutaMAX^TM^, 0.01% 2-ME, NEAA, FGF2	*MYOD1* constitutive expression (lentiviral system with EEF1A1 promoter)	Collagen I coating, α-MEM, 5% KOSR (7–10 days) **Markers** GE: *PAX7/3, MYOD1*, PE: n/a	**Markers** GE: *MYH2*, Ach receptor PE: MYH1, ultrastructure	Adherent	n/a	n/a	n/a
Rao et al., 2018^15^	H9, TRiPS, GM2525646, and Fucci	Matrigel^®^ coating with E8 medium	*PAX7* (dox-inducible lentiviral system)	Matrigel^®^ coating, with E6 media, 10 µM CHIR99021 (2 days); E6 media, 1 µg/mL dox (18 days) **Markers** GE: *MYHs* PE: MYOD1, PAX3, MYF5, MYOG, sarcomeric α-actinin, PAX7	logDMEM, 10% FBS, Fetuin (500X), hEGF (1000X), DE (1000X), pen (100 unit/mL), strep (50 µg/mL) until 80% confluence (for 2D culture) or 4 days (for 3D culture); logDMEM, N-2 Supplement (100X), pen G (100 unit/mL) (~ 2 weeks) **Markers** GE: n/a PE: sarcomeric α-actinin, ACh receptor, MYH, Ca^2+^ handling genes	Adherent vs 3D	Myogenic progenitors: FACS for iPAX7+ (GFP+)	ESt at 20% stretch (40 V/cm, 10 ms): twitch force per cross-sectional area of 0.8 mN/mm^2^ Ca^2+^ transients recording after 1, 2, 4 weeks (in vitro) upon ESt (10 ms pulse, 3 V/mm), and from muscle explants 7-15 days post-implantation	**Cell type:** progenitors (day 14 of differentiation) **Endpoint:** 15 days **Outcome:** the bundles were vascularized and remained functional after explant (injected dorsally or in TA muscle of NSG or nude mice)
Selvaraj et al., 2019^46^	Fibroblasts (PLZ, TC-1133, MNP-120, MNP-119)	Matrigel^®^ coating with mTeSR1	*PAX7* (dox-inducible lentiviral system)	IMDM basal medium, 15% FBS, 10% HS, 1% pen/strep, 1% GlutaMAX^TM^, 1% KOSR, 50 µg/mL AsAc, 4.5 mM MTG (altogether defined “myogenic medium”), 10 µM CHIR99021 (2 days); myogenic medium, 200 nM LDN193189, 10 µM (SB431542 (1 day); myogenic medium, 1 µg/mL dox (3 days); gelatin coating, myogenic medium, 1 µg/mL dox, 5 ng/mL FGF2 (4 days) **Markers** GE: *MEOX1, TCF15, PAX3, FOXC2* PE: n/a	KO DMEM, 20% KOSR, 1% NEAA, 1% GlutaMAX^TM^, 1% pen/strep, 10 µM SB431542, 10 µM DAPT, 10 µM DE, 10 µM PD0325901, 10 µM Forskolin (5 days) **Markers** GE: *MYOG, MYOD1, MYH2, MYH3, MYH8, MYH7* PE: MYH8, TTN, DES	EBs then adherent	Myogenic progenitors: FACS for iPAX7+ (GFP+)	3D construct with bovine fibrinogen, thrombin, and growth factor reduced Matrigel^®^ 3D. ESt at 20% stretch (10 ms, 0.5 Hz, or 20 Hz): twitch force of 0.4 mN.	n/a

Gene and protein symbols are in capital letters. Gene symbols are italicized.

^a^*Culture conditions and markers, 2-ME* 2-mercaptoethanol, *ACh* Acetylcholine, *ACTA1* Actin alpha 1, skeletal muscle, *AsAc* Ascorbic acid, *BSA* Bovine serum albumin, *CDH15* M-cadherin, *CHIR99021* GSK3β inhibitor, *CKM* Creatine kinase muscle isoform, *DAPT* Notch inhibitor, *DE* Dexamethasone, *DES* Desmin, *DMD* Dystrophin, *dox* Doxycycline, *DPC* Dystrophin-associated protein complex, *EBs* Embryoid bodies, *EEF1A1* Eukaryotic translation elongation factor 1 alpha 1, *ES* Embryonic stem, *ESt* Electrical stimulation, *FACS* Fluorescence-activated cell sorting, *FBS* Fetal bovine serum, *FGF2* Fibroblast growth factor 2, *GE* Gene expression, *GFP* Green fluorescent protein, *higDMEM* High glucose DMEM, *HS* Horse serum, *IGF-1* Insuline-like growth factor-1, *IMDM* Iscove’s Modified Dulbecco’s Medium, *iMEF* Irradiated mouse embryonic fibroblasts, *iMYOD1* Inducible MYOD1, *iPAX7* Inducible PAX7, *ITGA7* α7-integrin, *ITS* Insulin-transferrin-selenium, *KDR* Kinase insert domain receptor, *KO DMEM* Knockout DMEM, *KOSR* Knockout serum replacement, *LDN193189* BMP type I inhibitor, *L-glut* L-glutamine, *LGMD2D* Limb-girdle muscular dystrophy 2D, *LIF* Leukemia inhibitory factor, *LMNA* Lamin A/C, *logDMEM* Low glucose DMEM, *LY294002* Phosphoinositide 3-kinase inhibitor, *MEF2C* Myocyte enhancer factor 2C, *MEOX1* Mesenchyme homeobox 1, *MRF* Muscle regulatory factor, *MSGN1* Mesogenin 1, *mTeSR* cGMP feeder-free maintenance medium for human ESCs and iPSCs, *MTG* Monothioglycerol, *MYH* Myosin heavy chain, *MYOD1* myoblast determination protein 1, *MYOG* Myogenin, *n/a* not applicable, *Na pyr* Sodium pyruvate, *n/d* Not defined, *NEAA* Non-essential amino acid, *NSG* NOD scid gamma, *PAX* Paired box, *PD0325901* MEK/ERK pathway inhibitor, *PDGFRA/B* Platelet-derived growth factor receptor alpha/beta, *PE* Protein expression, *pen* Penicillin, *PSM* Presomitic mesoderm, *SB431542* TGF-β inhibitor, *SGCA/B/G* α/β/γ-sarcoglycan, *SSEA1* Stage-specific embryonic antigen 1, *strep* Streptomycin, *SYNE1* Spectrin, *T Brachyury, TA tibialis anterior, TPM2 Tropomyosin 2, TTN titin.*

**Table 2:** Non-transgenic approaches for human induced pluripotent stem cells skeletal muscle differentiation. The bibliographic research was performed until December 2020 using PubMed and SCOPUS databases. The search queries used for titles and/or abstracts starting from 2006 (year of the first report on iPSCs) were [(induced pluripotent stem cells) AND (skeletal muscle)]; [(skeletal muscle cell) AND (differentiation) AND (induced pluripotent stem cells)]; [(induced pluripotent stem cells) AND (myogenesis) OR (myogenic differentiation)]. Review articles, book chapters, and conference abstracts/papers were not included.

Ref.	iPSC origin and lines	Culture conditions in proliferation	Myogenic progenitor derivation^a^	Terminal differentiation^a^	Culture type	Myogenic cell selection	Functional tests	In vivo engraftment
Awaya et al., 2012^16^	Fibroblasts (01B6,201B7, 253G1, 253G4)	iMEFs with DMEM/F12, 20% KOSR, 1% NEAA, 5 mM NAOH, 100 µM 2-ME, 2 mM L-glut, 5 ng/mL FGF2	0.1% gelatin coating, DMEM, ITS-X, NEAA, GlutaMAX^TM^, 100 µM 2-ME (14 days) **Markers** GE: *PAX3, MYF5* PE: n/a	higDMEM, 10% FCS, 5% HS, NEAA, 100 µM 2-ME (98 days) **Markers** GE: *PAX3/7, MYF5, MYOD1, MYOG, DES, MYH2* PE: MYH	EBs	n/a	n/a	**Cell type:** myogenic progenitors (day 49 of differentiation) **Endpoint:** 4, 12, and 24 weeks **Outcome:** detection of human LMNA in the nuclei, and evidence of cell integration with the present muscle fibers. PAX7+ cells within the lamina rara (injected in cardiotoxin-treated TA muscles of NOD/Shi-scid/IL-2Rγ^null^ mice)
Sakurai et al., 2012^131^	Fibroblasts (201B7, 253G4)	n/a	Collagen I coating, α-MEM, 5% KOSR, 0.1 mM 2-ME (6 days); FACS purification; SF-O3, 5 mM LiCl, 10 ng/mL IGF-1, 10 ng/mL HGF, 10 ng/mL FGF2 (3 days) **Markers** GE: *T, PDGFRA, TBX6, MESP2, KDR* PE: n/a	SF-O3, 5 mM LiCl, 10 ng/mL IGF-1, 5 mM SB431542 (4 days); SF-O3, 10 ng/mL IGF-1, 5 mM SB-431542, 10 ng/mL HGF (7 days) **Markers** GE: n/a PE: MYH	Adherent	Myogenic progenitors: FACS for PDGFRA+/KDR‐	n/a	n/a
Hosoyama et al., 2014^63^	Fibroblasts (IMR90)	Matrigel^®^ coating with mTeSR1	EZ sphere in Stemline medium, 100 ng/mL FGF2, 100 ng/mL EGF, 5 ng/mL heparin sulfate (42–84 days). **Markers** GE: *PAX3, PAX7*, low *MYF5*, low *MYOD1*, low *MYOG* PE: PAX3, MYOD1, MYOG, MYH	Poly-L- lysine/laminin or Matrigel^®^ coating, DMEM, 2% B27 or 2% HS **Markers** GE: n/a PE: MYOD1, MYOG, MYH	EZ spheres	n/a	n/a	n/a
Chal et al., 2016^62^	hiPS11a, NCRM1, NCRM5	Matrigel^®^ coating with mTeSR1	Matrigel^®^ coating, DMEM/F12, 20 IU/mL pen, 0.02 mg/mL strep (2%), 1% NEAA, 1% ITS, 3 μM CHIR99021, 0.5 μM LDN193189 (3 days); same medium as before with 20 ng/mL FGF2 (3 days); DMEM/F12, 15% KOSR, 1% NEAA, 2% pen/strep, 0.1 mM 2-ME, 10 ng/mL HGF, 2 ng/mL IGF-1, 20 ng/mL FGF2, 0.5 μM LDN193189 (2 days) **Markers** GE: n/a PE: TBX6, PAX3	DMEM/F12, 15% KOSR, 1% NEAA, 2% pen/strep, 0.1 mM 2-ME, 2 ng/mL IGF-1 (4 days); same medium as before with 10 ng/mL HGF (18 days); DMEM/F12, 1% ITS, 2% pen/strep, 1% N-2 Supplement, 1% L-glut **Markers** GE: n/a PE: MYOD1, MYOG, PAX7, MYH2, TTN, DMD	Adherent	n/a	n/a	n/a
Iovino et al., 2016^61^	Fibroblasts	Matrigel^®^ coating with mTeSR1	STEM Diff Apel medium (STEMCELL Technologies), 10 ng/mL FGF2, 0.5 μM BIO, 20 M forskolin (7 days; FGF2, BIO, and forskolin added at days 1, 3, and 5) **Markers** GE: *PAX7, MYF5, MYOD1* PE: n/a	Matrigel^®^ coating, DMEM, 2% HS (29 days) **Markers** GE: *MYOG, MYH2* PE: n/a	EBs and then adherent	n/a	n/a	n/a
Wu et al., 2016^205^	DF19-9, WiCell	Matrigel^®^ coating with E8 essential medium	IMDM, 10% HS, 20% FBS, pen/strep, 3 μM CHIR99021 (4 days); same medium as before without CHIR99021, 10 ng/mL FGF2 (8 days) **Markers** GE: MYF5 PE: n/a	DMEM, 2% HS (5 days) **Markers** GE: n/a PE: MYH2	EBs and then adherent	Myogenic progenitors: FACS for MYF5+ (GFP+)	n/a	n/a
Shelton et al., 2016^58^	n/d	Matrigel^®^ coating with E8 medium	Matrigel^®^ coating, E6 medium, 10 µM CHIR99021 (2 days); E6 medium (10 days) **Markers** GE: *T, MSGN1, TBX6, PAX3, MEOX1* PE: T	StemPro-34 medium, 0.45 mM MTG, 5 µg/mL gent, 2 mM L-glut, 10.7 µg/mL transferrin, 5 ng/mL FGF2 (10 days); E6 media (15 days); DMEM/F12, 1% ITS, 1% N-2 Supplement, 0.01% gent (15 days) **Markers** GE: *MYOD1, MYOG* PE: PAX7, MYH2	Adherent	n/a	n/a	n/a
Swartz et al., 2016^60^	Fibroblasts	Vitronectin coating with TeSR-E8 medium	IMDM/F12, 5 mg/mL BSA, 100X lipids, 15 µg/mL transferrin, 450 µM MTG, 7 µg/mL insulin, 20 ng/mL FGF2, 10 µM LY294002, 10 ng/ mL BMP4, 10 µM CHIR99021 (36 hours); same medium as before without BMP4 and CHIR99021 (5.5 days); MB-1, 15% FBS (5 days) **Markers** GE: *T, PAX3, TBX6* PE: PAX3	DMEM, 2% HS (10 days); DMEM/F12, 1% N-2 supplement, 1% ITS (7–10 days) **Markers** GE: *MYOG, MYOD1, MYH, PAX3* PE: PAX7, MYOG, low TUBB3, MYH2, DES, TTN	Adherent	n/a	Spontaneous contraction at day 34 (0.1–0.3 contractions/s)	n/a
Maffioletti et al., 2018^21^	NCRM1, NCRM5, A13777	iMEFs with KO DMEM; 25% KOSR, 2 mM L-glut, 1 mM Na pyr, 100 IU mL pen, 100 mg/mL strep, 1% NEAA, 0. 2 mM 2-ME, 10 ng/ml FGF2^12^	Collagen I coating with SKM-01, 5% HS, 3 µM CHIR99021, 2 µM Alk5 Inhibitor, 10 ng/mL EGF, 10 µg/mL insulin, 0.4 µg/mL DE, 200 µM AsAc (10 days); SKM-02, 5% HS, 10 µg/mL insulin, 10 µg/mL EGF, 20 ng/mL HGF, 10 ng/mL PDGF, 20 ng/mL FGF2, 20 µg/mL oncostatin, 10 ng/mL IGF-1, 2 mM SB431542, 200 µM AsAc (8 days) **Markers** GE: n/a PE: PAX7, DES	SKM-03, 10 µg/mL insulin, 20 µg/mL oncostatin, 50 nM necrosulfonamide, 200 µM AsAc (7 days) **Markers** GE: n/a PE: MYH	3D	n/a	n/a	n/a
Sakai-Takemura et al., 2018^17^	253G4, 201B7, 409B2, 454E2	iMatrix-511 coating with mTESR1	iMatrix coating, DMEM/F12, 1% ITS, 3 μM CHIR99021, 0.5 μM LDN193189 (3 days); same medium as before with 20 ng/mL FGF2 (3 days); DMEM/F12, 10 ng/mL HGF, 2 ng/mL IGF-1, 20 ng/mL FGF2, 0.5 μM LDN-193189 (2 days); DMEM/F12, 15% KOSR, 2 ng/mL IGF-1 (4 days). Induction of EZ sphere culture in Stemline, 100 ng/mL FGF2, 100 ng/mL EGF, 5 µg/mL heparin sodium salt for at different time points (6–10 weeks)^63^ **Markers** GE: *T, TBX6, PAX3, PAX7* PE: MYOD, PAX7, MYOG	Collagen coating, DMEM, 10% FBS (4 weeks); DMEM, 10% FBS, 10 μM SB431542, and/or 10 μM DAPT **Markers** GE: *MRF4, MYH2, MSTN, MYH8, PAX7* PE: MYH2, MYOG, PAX7	Combination of adherent 2D culture and EZ spheres	Myogenic progenitors: FACS for CD57−/CD108− /CD271+/ERBB3+	n/a	**Cell type:** CD57−/CD108−/CD271+/ERBB3+ **Endpoint:** 1 month **Outcome:** myotubes were found positive for LMNA, SYNE1, DMD (injected in TA muscles of NSG-mdx^4Cv^ mice, daily injection of SB431542 to enhance cell differentiation for four days)
Van der Wal et al., 2018^18^	Fibroblasts (8 healthy donors)	iMEFs with DMEM/F12, 20% KOSR, 1% NEAA, 1% pen/strep/L-glut, 2 mM 2-ME, 20 ng/mL FGF2	DMEM/F12, 1% ITS, 1% pen/strep/L-glut, 3.5 µM CHIR99021 (5 days); same medium as before with 20 ng/mL FGF2 (14 days) **Markers** GE: n/a PE: PAX7	DMEM/F12, 1% ITS, 1% pen/strep/L-glut (16 days) **Markers** GE: RNA seq PE: MYH, TTN, α-ACTN	Ad﻿herent	Myogenic progenitors: FACS for c-MET+ /HNK-1-	Spontaneous contraction	**Cell type:** myogenic progenitors **Endpoint:** 1 month **Outcome:** cells were found positive for LMNA, SYNE1, DMD and contributed to new fiber formation. A small subset of LMNA+ cells were perivascular and PAX7+ (injected in injured TA muscle of NSG mice)
Al Tanoury et al. 2020^67^	NCRM1 with Venus reporter cassette in *PAX7*	Matrigel^®^ coating with mTeSR1	Matrigel^®^ coating, DMEM/F12, 20 IU/mL pen, 0.02 mg/mL strep (2%), 1% NEAA, 1% ITS, 3 μM CHIR99021, 0.5 μM LDN193189 (3 days); same medium as before with 20 ng/mL FGF2 (3 days); DMEM/F12, 15% KOSR, 1% NEAA, 2% pen/strep, 0.1 mM 2-ME, 10 ng/mL HGF, 2 ng/mL IGF-1, 20 ng/mL FGF2, 0.5 μM LDN193189 (2 days); DMEM/F12, 15% KOSR, 1% NEAA, 2% pen/strep, 0.1 mM 2-ME, 2 ng/mL IGF-1 (4 days); same medium as before with 10 ng/mL HGF (9 days); DMEM/F12, 1% ITS, 2% pen/strep, 1% N-2 Supplement, 1% L-glut **Markers** GE: n/a PE: TBX6, PAX3, MYOD1, MYOG, PAX7, MYH2, TTN, DMD	Skeletal muscle growth medium (SkGM-2, Lonza), 10 µM ROCK inhibitor (1 day); SkGM-2 (1-2 days); DMEM/F12, 2% KSR, 1 µM Chiron, 0.2% pen/strep, 1× ITS (1-2 weeks) **Markers** GE: n/a PE: PAX7, MYH2	Ad﻿herent	Myogenic progenitors (dissociated after 21 days of differentiation): FACS for PAX7^Venus^	n/a	**Cell type:** PAX7^Venus^ myogenic progenitors (day 21 of differentiation) **Endpoint:** 6–8 weeks **Outcome:** restoration of DMD expression, contribution to new fiber formation (hLMNA+/hSYNE1+) and repopulation of stem cell compartment (hLMNA+/PAX7+) (injected in TA muscle of Rag2−/− γc −/− or NOD; Rag1 −/−; Dmd^mdx-5Cv^ mice)
Baci et al., 2020^19^	Pericytes and fibroblasts	Geltrex matrix with E8 medium	Geltrex matrix, E6 medium, 1% ITS, 10 µM CHIR99021 (2 days); E6 medium, 1% ITS, 5 mM LiCl, 10 ng FGF2, 10 ng IGF-1, 50 µg/mL EVs from MT (4 days); cell splitting; collagen I coating, E6 medium, 1% ITS, 10 ng FGF2, 10 ng IGF-1, 50 µg/mL EVs from MT (10 days) **Markers** GE: *MSGN1, PAX3, PAX7, MYOD1, MYOG* PE: n/a	E6 medium, 1% ITS (10 days) **Markers** GE: *MYOG, MYH8, CKM, MYH* PE: MYH2, NCAM1, MYOD1, MYOG, MYH2	Ad﻿herent	n/a	n/a	**Cell type:** pericyte-iPSC-derived muscular cells (day 25 of differentiation) **Endpoint:** 20 days **Outcome:** sufficient engraftment and integration into regenerating muscle fibers revealed by the labeling of human-derived cells by immunofluorescence against human-specific lamin A/C antibody (injected in TA muscle of SCID-Beige/SGCA^null^ LGMD2D mice)
He et al., 2020^20^	Peripheral blood mononuclear cells	Matrigel^®^ coating with mTeSR1	STEM Diff Apel medium (STEMCELL Technologies), 10 ng/mL FGF2, 0.5 mM BIO, 20 mM forskolin (7 days) **Markers** GE: n/a PE: PAX7	Matrigel^®^ coating, DMEM, 2% HS (26 days) **Markers** GE: n/a PE: PAX7, MYF5, MYH4, DES, DMD	EBs and then ad﻿herent	n/a	n/a	**Cell type:** EGFP hiPSC-derived myogenic progenitors (day 14 of differentiation) **Endpoint:** 4, 8, and 12 weeks **Outcome:** restoration of DMD expression, contribution to new fiber formation and repopulation of stem cell compartment (systemic injection or in TA muscle of mdx mice)

Gene and protein symbols are in capital letters. Gene symbols are italicized.

^a^*Culture conditions and markers, 2-ME* 2-mercaptoethanol, *AsAc* Ascorbic acid, *BIO* 6-bromoindirubin-3′-oxime, GSK3β inhibitor, *BMP* Bone morphogenetic protein, *BSA* Bovine serum albumin, *CHIR99021* GSK3β inhibitor, *CKM* Creatine kinase muscle isoform, *DAPT* Notch inhibitor, *DE* Dexamethasone, *DMD* Dystrophin, *EBs* Embryoid bodies, *EGF* Epidermal growth factor, *EVs from MT* Extracellular vesicles released from differentiated myotubes, *FACS* Fluorescence-activated cell sorting, *FBS* Fetal bovine serum, *FCS* Fetal calf serum, *FGF2* Fibroblast growth factor 2, *GE* Gene expression, *gent* Gentamicin, *GFP* Green fluorescent protein, *HGF* Hepatocyte growth factor, *higDMEM* High glucose DMEM, *HS* Horse serum, *IGF-1* Insuline-like growth factor-1, *IMDM* Iscove’s Modified Dulbecco’s Medium, *iMEF* Irradiated mouse embryonic fibroblasts, *ITS* Insulin-transferrin-selenium, *KDR* Kinase insert domain receptor, *KO DMEM* Knockout DMEM, *KOSR* knockout serum replacement, *LDN193189* BMP type I inhibitor, *L-glut* L-glutamine, *LGMD2D* limb-girdle muscular dystrophy 2D, *LMNA* Lamin A/C, platelet-derived growth factor, *LY294002* Phosphoinositide 3-kinase inhibitor, *MEOX1* Mesenchyme homeobox 1, *MESP2* Mesoderm posterior bHLH transcription factor 2, *MSGN1* Mesogenin 1, *mTeSR* cGMP feeder-free maintenance medium for human ESCs and iPSCs, *MTG* Monothioglycerol, *MYF5* Myogenic factor 5, *MYH* Myosin heavy chain, *MYOD1* Myoblast determination protein 1, *MYOG* Myogenin, *n/a* Not applicable, *n/d* Not defined, *NEAA* Non-essential amino acid, *NSG* NOD scid gamma, *PAX* Paired box, *PDGF* Platelet-derived growth factor, *PDGFRA/B* Platelet-derived growth factor receptor alpha/beta, *PE* Protein expression, *pen* Penicillin, *SB431542* TGF-β inhibitor, *SF-O3* serum-free culture medium, *SGCA* α-sarcoglycan, *strep* Streptomycin, *T* Brachyury, *TA* Tibialis anterior, *TBX6* T-box transcription factor 6, *TTN* Titin, *TUBB3* Tubulin beta 3.

When the differentiation protocols are used to generate in vitro platforms for disease modeling and drug development, the in vitro induction of muscle progenitor differentiation is performed to create cultures of 2D myotubes, or three-dimensional artificial muscles with disease-specific hallmarks^21–24^. In some cases, their ability to depolarize or level of contractility is also tested^13,15,22,25,26^, to further validate their physiological relevance. Such information is useful also when using healthy cells for more advanced biomedical applications, such as the fabrication of iPSC-derived contractile biohybrid actuators^27^.

The main muscular diseases treated in clinical trials are genetic conditions such as muscular dystrophies and laminopathies, or tissue degeneration and VML due to injured or aged muscle. Some of the most studied muscular dystrophies are the Duchenne and Becker types, caused by mutations in the dystrophin (DMD) gene, which leads to a lack or a dysfunction of the related protein. Disease modeling with iPSCs can help treat these diseases, though there are challenges such as the high degree of clinical heterogeneity of the dystrophies^28^.

Nevertheless, few papers have highlighted the importance of biophysical stimuli for skeletal muscle differentiation^7,29,30^. In fact, during their embryonic development, cells are greatly influenced by their surroundings, which consist of complex interactions between biochemical and mechanical stimuli, distributed in space and time^31–34^.

To achieve a differentiated and functional skeletal muscle tissue starting from iPSCs, a controlled promyogenic environment thus needs to be created. This environment is not only ensured by the biochemical components, but also by a complex set of stimuli, resembling the ones available in an in vivo microenvironment.

This review is organized as follows. Section “Skeletal muscle tissue embryonic development and architecture” provides an overview of skeletal muscle development and differentiation, along with a description of the macroscopic and microscopic structure of the skeletal muscle in vivo as well as the various cell types. Section “Methods to induce skeletal muscle differentiation in iPSCs” details the two main methods for iPSC myogenic induction along with the main state of the art protocols and their in vivo application (Table 1 and Table 2).

Section “Biophysical stimulations for iPSC skeletal muscle differentiation” describes the biophysical stimuli that have been applied or could be applied to iPSCs to pursue skeletal muscle tissue engineering. Section “Challenges in the clinical translation of iPSC-derived skeletal muscle”, the challenges for iPSC translation in clinical settings are described. Finally, the section “Co-culture of skeletal muscle cells with muscle-resident phenotypes” focuses on multicellular cultures and the combinations of skeletal muscle cells with different muscle-resident cell types.

## Skeletal muscle tissue embryonic development and architecture

### Skeletal muscle development during embryogenesis

**Fig. 1:**
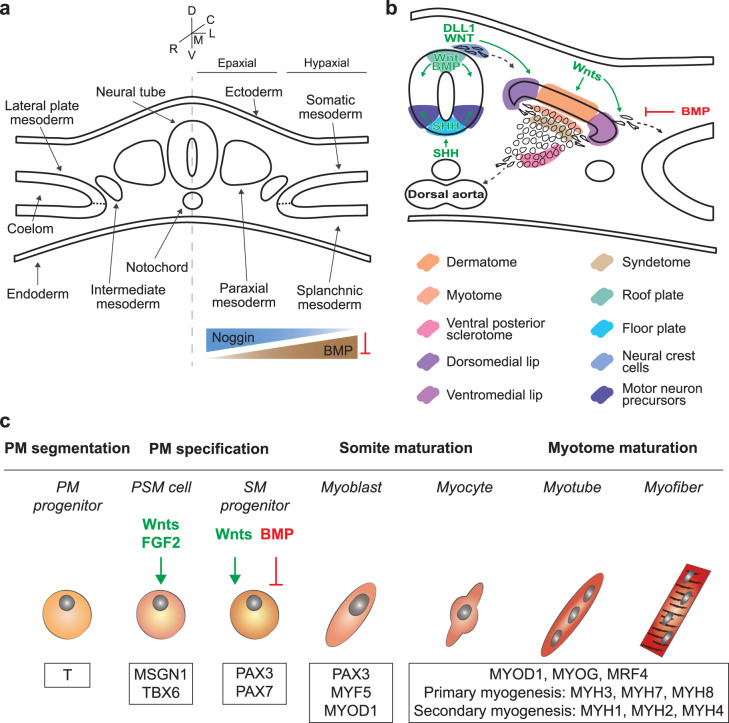
Skeletal muscle development. **a** Scheme of the mesoderm patterning along the mediolateral axis by gradients of specific signaling molecules, as Noggin and BMP. D dorsal, L lateral, M medial, V ventral, R rostral, C caudal. **b** Color-coded scheme of the differentiating somites and the surrounding structures during gastrulation and neurulation. Signaling molecules are indicated in green if acting as pro-differentiative actors, in red if they inhibit the differentiation process; dashed lines show paths of cell migration. **c** Representation of the differentiation process of skeletal muscle cells of the axial and limb muscles, starting from the paraxial mesoderm (PM) progenitors. Marker genes are shown in the bottom boxes, while the main signaling molecules are indicated in green if acting as pro-differentiative actors, in red if they inhibit the differentiation process. PSM presomitic mesoderm, SM skeletal muscle. Schemes adapted and modified from^35,206^.

Myofibers mainly derive from the mesoderm, which is one of the three germ layers created between the ectoderm and the endoderm during gastrulation, preceding the neural tube formation. The mesoderm undergoes a process of specification on the mediolateral axis, thanks to the action of Noggin and bone morphogenetic protein (BMP) signaling on the same axis. This then leads to the formation of the paraxial mesoderm, the intermediate mesoderm, and the lateral plate mesoderm (Fig. 1a).

Skeletal muscle cell development is a multistep process characterized by complex morphogen signaling, influences from the neural tube and notochord, and regulation of specific muscle-related genes. These processes are detailed in previous review articles^35–37^ and are shown in Fig. 1b, c.

Below is a summary of the key steps encountered by the differentiating cells, steps which are also recapitulated during in vitro iPSC differentiation.

Myogenic precursors of the axial and limb muscles originate from the segmented region of the paraxial mesoderm progenitors expressing the early mesoderm marker brachyury (T). The segments are called somites, which are transitory epithelial clusters of multipotent stem cells, located bilaterally to the neural tube. The different regions of the paraxial mesoderm are determined by gradients of Wnt signaling factors, fibroblast growth factor (FGF2), and retinoic acid, whose key target genes include mesogenin 1 (MSGN1) and T-box transcription factor 6 (TBX6) which are both presomitic mesoderm markers. Cells of the dorsal somatic region, the dermomyotome, then start expressing two paired-box transcription factors, PAX3 and PAX7, under the activation of Wnt signaling^37^.

Myogenesis is then divided into three stages: (1) primary myogenesis (with the formation of a scaffold of primary muscle fibers from embryonic progenitors); (2) fetal secondary myogenesis from PAX7+ cells (with the formation of MuSCs, and of secondary muscle fibers, fusing with each other and with primary fibers); and (3) adult-type myogenesis (muscle adaptation to applied stimuli and regeneration)^38^. Signals coming from the dorsal region of the neural tube (WNT1 and delta-like canonical Notch ligand 1, DLL1), specifically from the neural crest cells, activate the expression of muscle-specific transcription factors (i.e. the muscle regulatory factors), such as myogenic factor 5 (MYF5), myoblast determination protein 1 (MYOD1), myogenin (MYOG), and myogenic factor 6 (MYF6, also known as muscle regulatory factor 4, MRF4). MYOD1 and MYF5 are markers of terminal specification of the muscle lineage^39^, while MYOG controls the terminal differentiation of the myoblasts fusing with each other and forming multinucleated myotubes.

These primary myofibers, derived from dermomyotomal PAX3+ progenitors, start to express slow, embryonic, and perinatal myosin heavy chain (MYH) isoforms (MYH7, MYH3, MYH8 respectively) and myosin light chains^35^. In the secondary myogenesis, the central dermomyotome loses its epithelial features. PAX3+ cells then migrate towards the myotome, start expressing PAX7, and fuse together, as well as with the primary myofiber scaffold. They express fast MYH isoforms, such as MYH2 (MyHC-2A), MYH1 (MyHC-2X/D), MYH4 (MyHC-2B) (Fig. 1c).

Besides skeletal muscle cells, there are other cells that are key to muscle development, and which help achieve a mature muscle phenotype. Some of these cells have origins and timeframes similar to the skeletal muscle cells. The sclerotome is derived from the ventromedial somites under the myotome, with the cells undergoing an epithelial-mesenchymal transition and migrating ventrally. The sclerotome has three main progenitor zones: (1) the syndetome, which is located dorsally and that generates tendons; (2) the internal and lateral regions that form the joints, bones, and cartilage in the spine and the rib cage; and (3) the ventral posterior somites, endothelial precursor cells that form the dorsal aorta, the first intraembryonic blood vessel^40^.

### Cell and tissue organization

Skeletal muscle tissue is mainly composed of elongated multinucleated myofibers, which are specialized skeletal muscle cells. However, several other cell populations are present throughout the tissue, and are essential for muscle development and functioning: progenitor cells, cells from the connective tissues, cells of the vascular network, adipogenic cells, immune cells, and motor neurons. This section describes how these cells in adult muscle tissue are organized, while Section “Co-culture of skeletal muscle cells with muscle-resident phenotypes” gives an overview of their embryonic development in relation to muscle cells, and reports the results of co-culture experiments.

Myofibers are composed of packed myofibrils filling the whole sarcoplasm, i.e. the myofiber cytoplasm, which is enclosed in the myofiber membrane, called the sarcolemma. Myofibrils run along the length of the myofiber and have a modular architecture: the sarcomeres are repeated longitudinally and intercalated by structures called Z-disks.

**Fig. 2:**
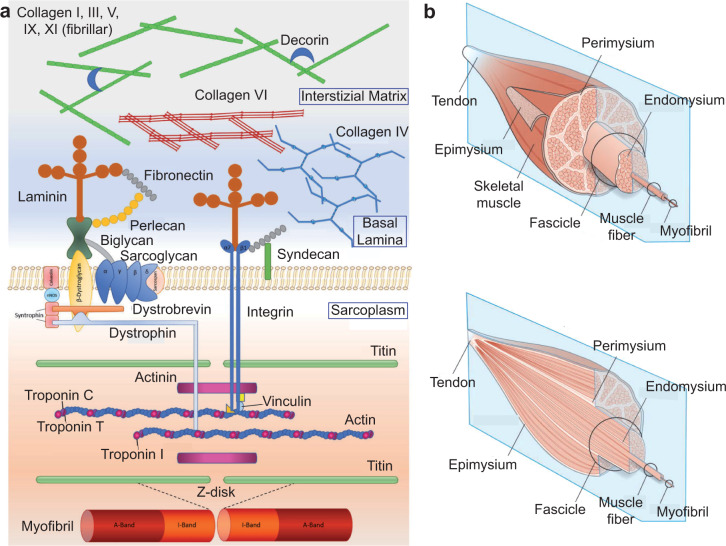
Skeletal muscle microenvironment and architecture. **a** The skeletal muscle cell contractile unit (the sarcomere, at the bottom) and its interface with the extracellular matrix. Image reproduced and adapted with permission from^43^. **b** Organization of the muscle tissue and the intramuscular connective tissue. Image reproduced and adapted with permission from^42^.

The sarcomere is the contractile unit, and by analyzing its ultrastructure, the filaments of myosin sliding on the actin filaments are observable, shortening the sarcomere and drawing nearer the two Z-disks. The sarcomere is composed of two halves of the I-band at the two extremities, the band that surrounds the Z-disk and is composed of actin filaments without myosin, and a central A-band, containing both actin and myosin filaments. Titin (TTN), actinin (ACTN), and desmin (DES), which are three of the proteins in the Z-disk, help to associate the myofiber cytoskeleton with the dystrophin-associated protein complex (DPC) and the α7β1 integrin (Fig. 2a). The DPC and the integrin are sarcoplasmic molecular complexes connecting the sarcomere with the extracellular matrix (ECM), specifically the basement membrane.

The basement membrane is part of the second most abundant tissue in the SM from a volumetric viewpoint, which is the connective tissue. Muscle connective tissues are important for muscle structural integrity and contractile force transmission, but they are also key in regulating muscle development. The intramuscular connective tissue is composed of continuous network structures, represented by the endomysium, the perimysium, and the epimysium. The musculoskeletal system also comprises other connective tissues, such as bones, cartilage, tendons, ligaments, and the adipose tissue^41^.

With regard to the components of the intramuscular connective tissue, the endomysium is wrapped around a single muscle cell, the perimysium surrounds bundles of muscle cells, while the epimysium is located around the whole muscle (Fig. 2b). The intramuscular connective tissue is composed of dispersed cells in an ECM of proteoglycans rich in fibrillar protein such as collagen and elastin. Parallel bundles of type I collagen confer tensile strength and rigidity to all three layers; type III collagen confers elasticity to endo- and perimysium; type IV collagen, with its helical structure, can be found in all three layers, but it is concentrated mostly in the basement membrane.

Knowledge of their specific architecture, protein, and cellular composition is impaired by a lack of standardized and systematic approaches in the analysis protocols^42^.

The endomysium (0.2–1.0 µm thick) is a mesh of quasi-randomly orientated collagen fibers. It interacts with the sarcolemma through the 50 nm-thick basement membrane, mainly composed of type IV collagen and laminin, which in turn interacts with the two abovementioned sarcolemmal structures: the DPC and α7β1 integrin^43^. The perimysium of the different muscles varies in thickness, with a small amount of elastin next to collagen bundles laying at ±55° to the muscle fiber direction at rest^41^. Lastly, in the epimysium, collagen bundles are oriented similarly to the perimysium or are parallel to the muscle long axis, depending on the muscle type.

On the other hand, tendons attach muscles to bones, thanks to a continuum with the intramuscular connective tissue. They are composed of an ECM mainly made of crosslinked type I collagen fibrils (which can endure strong tensile forces), and tenocytes, a fibroblast subtype.

The skeletal muscle tissue has a high metabolism and therefore needs continuous nutrition, which is enabled by a thick network of capillaries wrapping every individual muscle fiber. Pericytes, together with endothelial cells CD31+ and the basal lamina, form the walls of the smallest division of the vascular system, i.e. the micro-vessels. Pericytes are present in skeletal muscle tissue with a ratio of approximately 1:10 with respect to endothelial cells^44^. They affect the migration, proliferation, and contractility of the capillary endothelial cells.

Skeletal muscle voluntary contraction is controlled by the motor neurons, which interact with the muscle cells at the neuromuscular junction. The motor neurons are divided into upper and lower. The upper motor neurons have the cell body in the cerebral cortex, while the lower are located in the spinal cord and the brainstem. Lower motor neurons are in direct contact with the controlled muscles, and are further subdivided into other groups according to the innervated target. The lower neurons include somatic motor neurons which specifically innervate skeletal muscles^45^. The lower spinal motor neurons have been studied the most, and are the longest cells in the body.

## Methods to induce skeletal muscle differentiation in iPSCs

### Transgenic approaches for iPSC-derived skeletal muscle cells

A successful approach for the myogenic differentiation of iPSCs is based on the transient overexpression (e.g., with mRNAs, non-integrative vectors) or stable genome integration (e.g., with integrative vectors) of muscle-related transcription factors such as *MYOD1*, *PAX3*, and *PAX7*. Some of the most relevant protocols are described in Table 1.

Different systems can be used to stably integrate a specific cDNA sequence in the iPSC genome for gene overexpression, such as the PiggyBac transposon system. The insertion of a doxycycline-responsive element in the transposon vector allows gene overexpression to be controlled by antibiotic addition in the culture medium. The stable integration and subsequent expression of the exogenous cDNA sequence can be tracked at the beginning of the differentiation protocol. Proliferating iPSCs can be enriched by manual clone selection or by fluorescence-activated cell sorting (FACS) for the successful transgenic expression of *MYOD1* or PAX3/7 fused to a fluorescent reporter gene, such as green fluorescent protein (GFP) construct^11,15,46^ or mCherry^13,25^.

Only a few protocols follow these enrichment procedures at this stage of differentiation, which may put mechanical stress on the differentiating cells (e.g., FACS).

The use of muscle-related transcription factor overexpression started with the initial demonstration that fibroblasts can be converted into muscle cells by using 5-azacytidine, an aspecific demethylating agent, which also targets the *MYOD1* locus^47^. *MYOD1* is a master regulator for myogenic specification, and *MYOD1* expression is crucial for myogenic induction. *MYOD1* also plays a role in myogenic commitment in non-muscle cells^48^ and ESCs^49^. Regarding iPSCs, the coexpression of *MYOD1* and *SMARCD3* (*BAF60C*), a chromatin remodeler, is needed. Albini et al. demonstrated that the absence of *SMARCD3* in proliferating iPSCs impairs the activation of myogenic genes mediated by *MYOD1*^50^. *MYOD1*-reprogrammed iPSCs using the PiggyBac system resulted in 70-90% of myogenic cells after five days of differentiation^13^. *MYOD1* overexpression has also led to the establishment of in vitro systems for high-throughput drug screening^51^.

Another strategy for direct myogenic induction consists of overexpressing transcription factors that precede *MYOD1* expression during embryonic development, namely *PAX3* and *PAX7*. *PAX7*-reprogrammed iPSCs can generate myogenic progenitors CD29+/CD44+/CD56+ when cultured in the form of embryoid bodies (EBs). EBs are three-dimensional cell aggregates, successfully used for 2D or 3D tissue modeling, with a size of a few hundred micrometers. They mimic early human embryogenesis by recapitulating the three embryonic germ layers. In one study, myogenic precursors derived from *PAX7*-reprogrammed EBs were engrafted for two months into a dystrophic mouse muscle, and restored dystrophin (DMD) expression and improved the muscle-generated force^11^. EB formation is strongly influenced by various parameters (e.g. culture medium conditions, cell number), which can lead to non-specific differentiation or core necrosis. Therefore, to standardize and scale up the procedure, various methods have been developed, such as the use of bioreactors or non-adhesive microwells^52,53^.

Protocols based on transgenic approaches are characterized by an initial differentiation phase towards a mesodermal phenotype. This phase is followed by the consequent induction of the transcription factor overexpression by introducing the specific antibiotic in a nutrient-rich medium, enriched with between 2 and 20% serum. These initial phases are followed by terminal differentiation in the presence of a medium with a low serum concentration (2–5%), in the presence of insulin stimulation (N-2 supplement or insulin-like growth factor-1, IGF-1). The differentiation efficiency is high and provides terminally differentiated myotubes MYH+/TTN+/DES+ in 10–15 days.

In some cases, *MYOD1* overexpression from the beginning of the differentiation protocol means that early embryonic differentiation can be bypassed, thus starting the myogenic induction from myoblast-like progenitors^13,24,25,51^. This means that the cells cannot be used to study early myogenesis processes. It is also not entirely clear how well the reprogrammed cell population phenotypically and genotypically represents a mature skeletal muscle tissue, since these cells do not follow the very defined transitions through all the myogenic developmental stages.

A few studies have coupled the development of a differentiation protocol and the functional evaluation of muscle fiber contractility or depolarization ability upon biomimetic stimuli, such as electrical^13,25^ or chemical stimulation by acetylcholine^26^. Rao et al. reported a functional 3D muscle bundle in vitro, thus bypassing EB formation^15^. They induced *PAX3* expression in iPSCs and generated differentiated 3D fascicles in two weeks. These fascicles produced a force (~0.8 mN/mm^2^) similar to primary myobundles, whose functionality was maintained even after the two-week engraftment. Rao also reported a short in vivo observation, which revealed host vascularization of the construct. However, the functional analyses of the bundle provided key information on the potential of the construct.

From a therapeutic viewpoint, the induction of *PAX3/7* in iPSCs can generate muscle progenitor cells that populate the stem cell niche when implanted in vivo, and then repair injured muscles^54^.

Furthermore, the implantation of PAX7-induced myogenic progenitors led to an increase in the tetanic, absolute, and specific muscle force in NSG mice^11^. A follow-up study showed that starting from PAX7-induced myogenic progenitors and enriching for ICAM1+/integrin α9β1+/SDC2+, a considerable regenerative capacity can be obtained in vivo. In fact, 10 months post-transplantation, the triple-positive cells replenished the satellite cell pool and generated new fibers, and no teratoma formation was observed^55^.

*MYOD1*-reprogrammed iPSCs cannot replenish the muscle stem cell niche, as they do not express *PAX3* or *PAX7* and thus do not show the regenerative potential of adult stem cells^54^. Conversely, using cells that are slightly different from classic PAX7+ muscle progenitors, *MYOD1*-reprogrammed iPSC-derived mesangioblast-like cell transplantation in Sgca^null^/scid/beige mice fused with the host fibers, reconstitute the DPC and repopulate the regenerative pool of the alkaline phosphatase+ pericytes^12^. However, too few studies have assessed the potential of myogenic progenitors generated with transgenic approaches to repopulate the stem cell niche.

Despite successful long-term studies on mouse models, the random integration of the overexpressed gene due to the use of integrative vectors may limit the translation of this technology to the clinic. In fact, the insertion of exogenous DNA in a random locus in the genome may cause genomic instability, thus interfering with cellular processes. Alternative transient approaches would be worth investigating such as non-integrative vectors, mRNA transfection, or transduction of recombinant proteins, even though they lead to a less efficient differentiation (~40%)^56^.

### Non-transgenic approaches for iPSC-derived skeletal muscle cells

Directed differentiation of iPSCs just by using defined culture conditions aim to recapitulate in vitro the multi-step differentiation process of the in vivo development. This is a spatio-temporal controlled concert of molecular and cellular processes (see Section “Skeletal muscle tissue embryonic development and architecture”). The most relevant protocols are described in Table 2, which exploit a sequential addition to the culture medium of different morphogens, growth, and differentiation factors, responsible for cell proliferation, migration, and differentiation in vivo.

Preliminary attempts to engineer skeletal muscle in vitro used laborious protocols that started with EB formation but ended in low-efficiency myogenesis^16,57^. Consequently, protocols on 2D cultures were developed. Initial steps regarded paraxial mesoderm induction and the formation of presomitic mesoderm progenitors (MSGN1+/TBX6+/PDGFRA+) by Wnt activation, using GSK3β inhibitors such as CHIR99021^15,17,21,58–60^ or 6-bromoindirubin-3′-oxime^61^. The simultaneous inhibition of BMP signaling is crucial as it controls the mediolateral identity of the paraxial mesoderm, with inhibitors such as LDN193189^17,62^ or SB431542^17,21^. The cells start expressing *PAX3*, an anterior presomitic mesoderm marker, and they can be used to generate a great number of myotubes^59^. Later, during myotome formation, Wnt signaling is still critical for dermomyotome specification and *PAX3*/*7* expression. Other factors can then be added to the differentiating myoblasts, such as FGF2, which acts on dermomyotome progenitors (*PAX*+) and promotes myoblast proliferation; hepatocyte growth factor (HGF), which supports myoblast migration and the expression of MYF5; and insulin, which activates Wnt signaling and thus promotes early myogenesis^35^.

As a final step, serum-free or low-serum media (frequently with N-2 supplement)^62^ promote terminal differentiation and spontaneous activity of skeletal myotubes^60,63^.

Regarding additional factors during terminal differentiation, IGF-1 promotes cell fusion and terminal myogenesis in *MYOD*+ myocytes, the first postmitotic cells. The addition of dexamethasone, a synthetic member of the glucocorticoid class, also plays a role in the terminal differentiation by inducing the synthesis of sarcolemmal and structural proteins, thereby enhancing sarcomeric organization^15,21^. Baci et al. proposed an intriguing approach: they used extracellular vesicles derived from C2C12 differentiated myotubes in synergy with CHIR99021 to derive MYH2+ myotubes^19^. The combination of CHIR99021 and extracellular vesicles, containing various myogenic factors, resulted in a more differentiated population compared to CHIR99021 alone, as highlighted by a greater expression of the myogenic markers *MYH2* and the creatine kinase muscle isoform.

Non-transgenic protocols usually lead to a heterogeneous cell population that is, in some cases, enriched by FACS for various myogenic progenitor markers, thus trying to level out the population phenotype. Cell population enrichment may also help to remove non-differentiated cells, whose presence leads to non-fully differentiated in vitro culture and teratoma formation in vivo. The progenitor population is enriched by positively selecting the cells for markers such as VCAM1 (CD106, SM/C2.6), CD34, NCAM1 (CD56), CXCR4 (CD184), and others that enrich the population for certain myogenic phenotypes with different efficiencies (extensively reviewed by Tey et al*.*^64^). Of the various markers, ERBB3, a receptor tyrosine-protein kinase, is a much better surface marker for myogenic selection from PAX7+ muscle progenitors in a directed differentiation^17,65^ than the frequently used combination of CD56 and CD82. ERBB3+ cells have a similar engraftment efficiency to fetal myogenic progenitors in mdx-NSG mice, and can restore approximately 10% of the myofibers^65^. However, ERBB3 is also enriched in Schwann cell progenitors, thus additional markers for myogenic population purification probably need to be identified^66^.

Although considerable effort has been made to identify cell markers that can be used to enrich the cell population of myogenic progenitors, there is still no unique marker or combination of markers that can be used to identify iPSC-derived myogenic progenitors. Given the importance of reducing the heterogeneity of the population in these experiments, several research groups are currently comparing the expression of surface markers between different differentiation protocols, with controversial results on marker expression in myogenic progenitors, maybe also due to the use of different cell lines and different culture conditions^64,66^.

Compared to protocols based on a transgenic approach, directed differentiation protocols are longer (mature myotubes TTN+/DES+ emerge after at least 25-50 days) since the cell population is guided through several differentiation stages, mirroring the developmental ones (Fig. 1b, c). Protocols can last up to 10 weeks if they combine 2D culture with EBs^17,63^, sometimes leading to a population with a low fusion index and level of maturity. Nevertheless, several protocols have successfully obtained muscle progenitors in vitro, which can differentiate into contractile myotubes TTN+/DES+, and show regenerative capacity also in vivo.

CD57−/CD108−/CD271+/ERBB3+ cells generate new muscle fibers after implantation, but need a daily injection of TGF-β inhibitor to enhance and sustain cell differentiation, which otherwise can be poor^17^. In vivo studies show that myogenic progenitors generate new muscle fibers even up to six months post-implant^16,67^. They can also restore DMD production in NSG-mdx^4Cv^ mice^18,20,67^, and repopulate the stem cell compartment^16,18,20,67^. Nevertheless, to achieve a high number of PAX7+ muscle progenitors for use in clinical applications, the problem of expanding them remains, since PAX7+ cell proliferation potential and stemness are impaired when subcultured in vitro^68^.

Differentiating iPSCs could also be used in clinical trials and studied at different developmental points. Furthermore, due to the absence of any gene insertion, iPSCs differentiated by directed myogenic induction have shown stability for up to four months in vitro, with many MYH+ cells surrounded by PAX7+ progenitors^60^. These protocols can generate a great number of myogenic progenitors, which can also be subcultured and subsequently differentiated^19,58,62^.

Moving beyond classical in vitro protocols, a new trend is potentially opening up a new frontier in the genesis of muscle progenitors^69^. Chan et al. isolated ITGA7+/VCAM1+ myogenic progenitors from iPSC-derived teratomas generated in NSG-mdx^4Cv^ mice, demonstrating their ability to pervasively regenerate DMD+ fibers after one month. The cells generate PAX7+ progenitors that differentiate into MYH+ myotubes when excised and differentiated in vitro, and no signs of teratomas were seen after 12 months post-implant.

## Biophysical stimulations for iPSC skeletal muscle differentiation

### Electromagnetic stimulation

During in vivo muscle development, motor neuron precursors electrically stimulate the cells they are connected to, thus promoting myogenesis. This takes place in mature skeletal muscles, whose healthy maintenance depends, among other factors, on innervation which ensures the differentiation of MuSCs and the conversion of MYH into the fast isoforms. Electrical stimulation is thus a possible biophysical tool for skeletal muscle tissue engineering.

Exogenous electrical stimuli typically affect the behavior of voltage-gated ion channels on the cell membrane. This results in the migration of a series of ions such as Ca^2+^ from their respective stores to the intracellular environment, eventually leading to a contraction of the sarcomeres, but it also activates intracellular pathways (e.g., MAPK/ERK or PI3K/AKT/mTOR) that ultimately affect the expression of certain genes and proteins.

Electric fields can promote myogenesis in C2C12 murine myoblast models and human primary muscle progenitor cells. The typical stimulation protocols (intermittent or continuous) are based on cells positioned between two or more flat electrodes, using frequencies ranging from 1 to 20 Hz, intensities ranging from 2 to 40 V/cm, and pulse widths from 2 to 20 ms. The main effect of such stimuli is a higher expression of DMD and fast MYH isoforms, and a more efficient organization of the sarcomeric units^70–73^.

The beneficial effects of electrical stimulation on the cardiac differentiation of human iPSCs are well known, thanks to the activation of Ca^2+^/PKC/ERK pathways and the expression of several genes leading to improved maturation of myocardial tissues^74–76^. In these studies, the electrical stimulus typically consisted of a brief (acute) stimulation (~5 min per day) at a frequency of 1–5 Hz, an intensity of 0.2–5 V/cm, and 1 ms pulse width.

With regard to human iPSC-derived skeletal muscle cells, Rao et al*.* obtained interesting results via transient overexpression of PAX7 in paraxial mesoderm cells differentiated from human iPSCs^15^. Rao stimulated the constructs with a 40 V/cm, 10 ms long electrical pulse at 5, 10, 20, and 40 Hz using a pair of platinum electrodes. However, such stimulation patterns were only applied at the endpoint to assess the tissue twitch force and thus to evaluate its maturation level. There are currently no extensive studies on the effects of acute/chronic electrical stimulation on the differentiation of iPSCs into skeletal muscle tissue. More studies are needed to verify the precise effects of electrical stimulation (and of its tunable parameters) in inducing specific phenotypes, for this cell type. Mild electrical stimulation appears to strongly influence ESCs to assume a neuronal fate. This influence peaked when 10 V were applied, but was less evident when lower or higher voltages were used^77^. Although iPSCs and ESCs show similar pluripotency, directly transferring the findings on one cell type to another is not straightforward. Systematic studies are thus needed on iPSCs to clarify whether electrical stimulation boosts the expression of mesodermal or ectodermal markers.

Similar considerations apply to magnetic stimulation. The exact mechanism of how magnetic fields trigger muscle restoration or differentiation has been not been deeply studied in vitro and the majority of these studies focus on rehabilitation procedures in mice and humans^73^. However, magnetic fields from 80 mT to 10 T can boost myofiber differentiation and alignment. Like electrical stimulation, magnetic stimuli have never been used to boost iPSC differentiation in the skeletal muscle phenotype.

Although electromagnetic stimulation deserves to be explored more in-depth the electromagnetic stimuli currently used are often variable and the set-ups to generate them are not standardized. Proper control of the energy dose delivered is needed, as well as a systematic screening of different experimental conditions in order to identify the most appropriate ones to tune cell behavior. Moreover, not all biological laboratories have a direct access to such technologies. Finally, when using these systems, undesired phenomena may occur such as water electrolysis, ion release, electrode degradation, and electrogenic damage to cells^78^. However, some of these effects can be reduced, for example by using alternated currents and pulsed stimulation and by protecting electrodes with functional coatings^79^.

### Mechanical stimulation

Skeletal muscle is very responsive to mechanical stimuli, as demonstrated for instance by the growth of muscle cell volume due to myofibrillar hypertrophy following periodic weightlifting. On the other hand, a long period of immobility may end up in muscle atrophy, and one of the reasons is the lack of mechanical stimulation. Mechanical stimuli are therefore essential to maintain the adult skeletal muscle structure, but they are also a vital stimulus during muscle development and regeneration^80^. Mechanical stimulation in vitro has thus been exploited as a biomimetic input to enhance the maturation and contractility of engineered muscles.

Regarding the general response of cells to mechanical stimulation, at the microscopic level, cells adhere to a certain substrate via adhesion molecules (e.g., dystroglycan, integrins), which link the cytoskeleton to the external environment. The cell membrane has several mechanosensors, among which integrin receptors and mechanosensitive channels such as stretch-activated ion channels (SACs), which are all responsible for physical force transduction. Mechanical force transduction mainly passes through changes in protein conformation due to the interaction with a specific ligand or due to microenvironment perturbation, with the subsequent activation of intracellular signal transduction pathways affecting the cell behavior.

The activation of integrin receptors and subsequent clustering in focal adhesions (the major tension sensors), recruits several signal transducers, e.g. cytoskeletal proteins, kinases, and phosphorylases. SACs, instead, are influenced by the change in membrane tension which increases the probability to open channels, thus leading to an ion influx^81^.

**Fig. 3:**
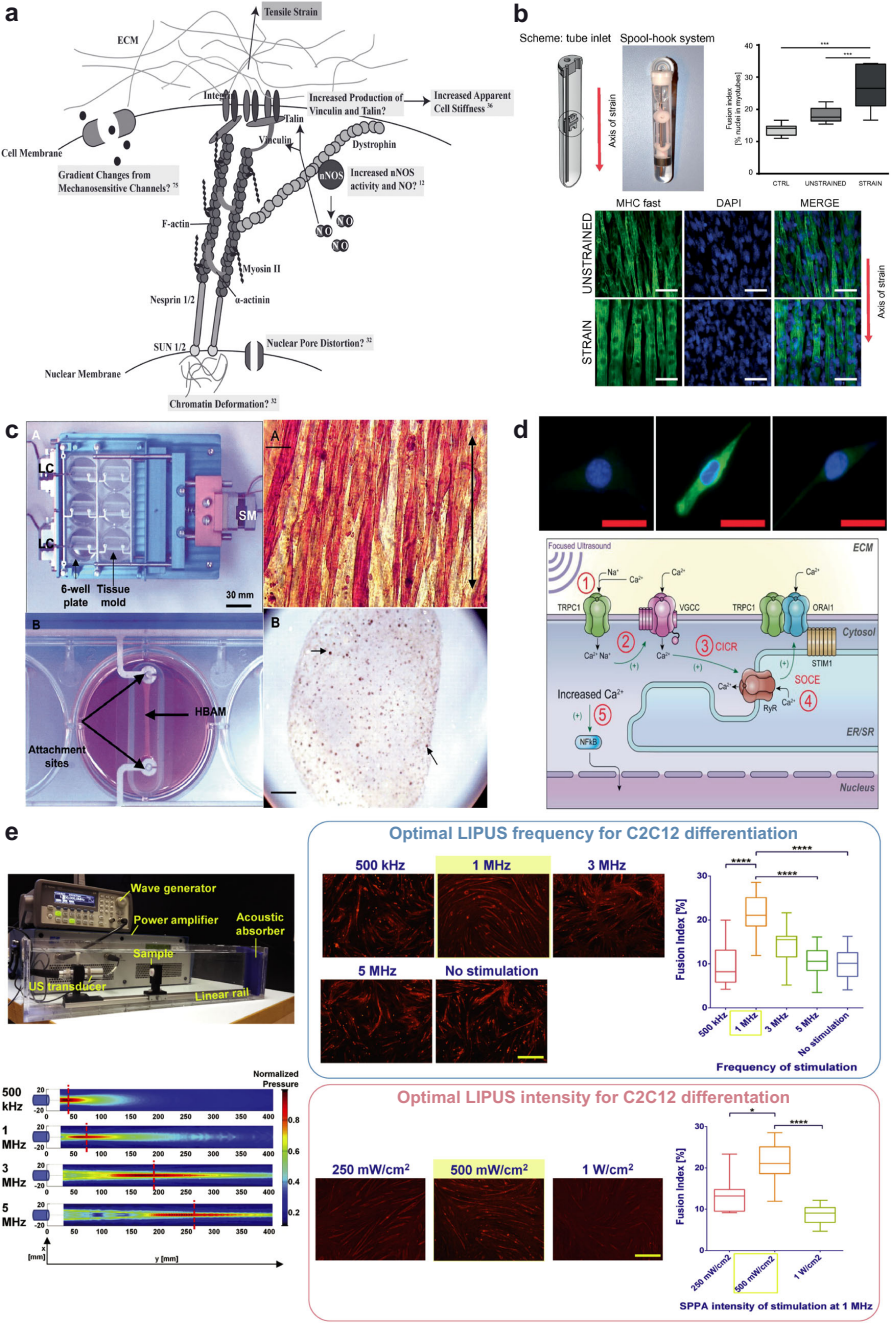
Effects of mechanical stimuli on skeletal muscle cells. **a** Representation of a possible mechanism responsible for myogenic differentiation due to tensile strain. ECM extracellular matrix, nNOS nitric oxide synthases, NO nitric oxide. Image reproduced and adapted with permission from^83^. **b** Top left: the MagneTissue bioreactor system for static mechanical stimulation of a fibrin ring. Top right: quantification of the fusion index at day 9. **p < 0.01; ***p < 0.001. Bottom: unstrained and strained myofibers from the fibrin rings after static mechanical stimulation and 6 days of differentiation. Cells are stained for MYH fast (green) and nuclei (DAPI, blue). Scale bars: 50 μm. Images reproduced and adapted with permission from^89^. **c** Mechanical cell stimulator based on a stepper motor (top left), moving one attachment site for each well (bottom left). *Top right*: construct stained for sarcomeric myosin (brown) after two weeks in culture. The black arrow indicates the axis of strain. Scale bar: 20 μm. Bottom right: cross-section of the 3D construct. Scale bar: 100 μm. Image reproduced with permission from^94^. **d** Bioeffects triggered by HIFU on murine muscle precursors (C2C12 cells): top images show cells immunostained for COX-2 (green) and nuclei (blue) 24 h post-treatment. HIFU upregulated COX-2; upregulation was blocked when cells were loaded with 1,2-bis(o-aminophenoxy)ethane-N,N,N’,N’-tetraacetic acid tetra(acetoxymethyl) ester (BAPTA-AM), a cell-permeable Ca^2+^-specific chelator, before HIFU stimulation. Scale bars: 10 μm. Bottom: scheme of intracellular Ca^2+^ signaling generating ultrasound bioeffects. Through a series of steps, ultrasound determines the activation of nuclear factor κ B (NFκB) that generates molecular responses (including COX-2). TRPC1 transient receptor potential cation channel subfamily C member 1, VGCC voltage-gated Ca^2+^ channel, CIRC Ca^2+^-induced Ca^2+^-release, SOCE store-operated Ca^2+^ entry, RyR ryanodine receptor, STIM1 stromal interaction molecule 1, ORAI1 Ca^2+^ release-activated Ca^2+^modulator 1. Images reproduced with permission from^104^. **e** Engineered ultrasonic set-up, provided with quantitative pressure maps for different transducers working at different frequencies (left) and results obtained on C2C12 cells for the different stimulation regimes in terms of myotube development (right). The optimal frequency and the optimal intensity guaranteeing the highest fusion indexes were identified. Scale bars: 500 μm. *p < 0.05, ****p < 0.0001. Images adapted and reproduced with permission from^108^.

The role of mechanical stimulation in myogenesis has been explained by three main hypotheses (Fig. 3a)^82,83^. First, the mechanically-triggered signaling may directly activate the transcription of myogenic transcription factors such as *MYOD1*, due to the direct stimulation of the nuclear membrane^84^. Second, the activation of mechanosensitive channels such as SACs can lead to a Ca^2+^ influx, which coordinates the expression of terminal differentiation markers downstream of *MYOG* expression via the activation of Ca^2+^-dependent signaling^85^. Third, mechanical cues may activate the neuronal nitric oxide synthase pathway, which can amplify the strain stimulation by nitric oxide release^86^ and prevent muscular atrophy by AKT serine/threonine kinase 1 (AKT1/PKB) phosphorylation^87^.

These pathways (isolated or in synergy) activate different intracellular responses, leading to the improvement of myoblast fusion, myofiber and sarcomere organization^88^, cell alignment along the principal axis of strain^89^, an increase in cross-striation of the muscle cells, and support for terminal differentiation by enhancing the switch from embryonic to adult *MYH* isoforms^90^.

The main mechanical stimuli that can be exploited for skeletal muscle tissue engineering are tensile strain, ultrasound, and altered gravity. These stimuli are discussed in the following subsections.

#### Tensile strain

A strain stimulus has several parameters, such as strain frequency, amplitude, duration, and resting period. Uniaxial tensile strain has a more powerful myogenic effect compared to biaxial tensile strain, probably due to its biomimetic action, while static and cyclic strain may have different effects by interfering with distinct pathways^83^.

The contribution of mechanical strain to myogenic progenitors/myoblasts differentiation has been reviewed by Wang et al*.*^91^. The strain regimes vary but positive effects have been reported on the myogenic outcome following different stimulation^33,83,92^. Heher et al*.*^89^ applied 6 h/day of 10%-static strain starting from the beginning of the C2C12 differentiation protocol, recapitulating the native growth of the musculoskeletal apparatus^93^, and thus enhancing the myogenic outcome by improving the expression of myogenic differentiation markers (Fig. 3b).

The role of cyclic strain in late differentiation stages (on formed myotubes), driving cell hypertrophy and maturation^94^ (Fig. 3c) has been extensively investigated. These two stretching regimens have been combined in a biomimetic protocol. In some studies, the constructs initially underwent a static pre-strain, which is a hallmark of embryonic development, followed by cyclic stretching, typical of the postnatal physical activity^94,95^.

A 10–15% strain amplitude seems to foster myogenic differentiation^83,91,96,97^ and a stimulation frequency of 0.5 Hz induces cell alignment^97^. However, there have been inconsistent results showing that mechanical strain may also impede myoblast withdrawal from the cell cycle. These contradictory conclusions remain to be elucidated and may perhaps be due to the cell source, differentiation status, culture conditions, and lack of standardized protocols^91^. One key issue is the difficulty in measuring the real entity of the transmission efficiency of the tensile stimulus from the 2D or 3D substrate directly to the cells. This factor depends on the cells’ interaction efficiency with the substrate, and the parameters of the stimulation protocol may likely be specific to the cell type used and the biomaterial chosen.

To ensure that the displacement reported at the macroscopic level is the same microstrain experienced by the cells, polystyrene microspheres (500 nm diameter) mixed in the matrix could possibly be tracked^98^. Nevertheless, these differences need to be more deeply characterized to ensure greater reproducibility of the experiments.

Despite many studies on the effect of strain-based mechanical stimuli on different myogenic cells, iPSC-derived skeletal muscle cells have not been investigated. Some research groups have studied the anti-pluripotency effect on undifferentiated iPSCs (down-regulating pluripotency markers after 12 h of cyclic strain^99^), and another group focused on the effects on tenogenic differentiation^100^. However, most reports on iPSC mechanical stimulation regard cardiomyocyte differentiation protocols^101^.

#### Ultrasound

Ultrasound is a form of acoustic energy at frequencies greater than 20 kHz. It is based on longitudinal waves that advance in a medium through the alternation of compression and rarefaction areas, thus delivering mechanical (and in some cases thermal) energy at the target. The two main ultrasound stimulation modalities in the biomedical field are high-intensity focused ultrasound and low-intensity pulsed ultrasound.

In high-intensity focused ultrasound, the ultrasound beam is focused on a small focal target, reaching high intensities and intense heat. This modality is mostly used to thermally ablate a tissue portion (e.g., a tumor)^102^, but it is also exploited in neurosurgery, blood-brain barrier permeabilization, and drug delivery^103^.

High-intensity focused ultrasound can also trigger non-destructive (regenerative) effects. Burks et al*.* demonstrated that focused ultrasound waves (pressure = 4 MPa) increased the tropism of murine muscle precursors (C2C12 cells) by altering molecular microenvironments through cyclooxygenase-2 (COX-2)-dependent pathways, in particular by indirectly activating voltage-gated Ca^2+^ channels (Fig. 3d). This activation was mainly mechanical, although temperature also played a role as it rose by 1.2 °C during the stimulation^104^. In another study, high-intensity focused ultrasound and the consequent mild hyperthermia produced were used to activate transgene expression, by exploiting a heat-activated gene expression system^105^. The feasibility of this approach was demonstrated on fibroblasts, but it could also enhance the transgenic methods described in Section “Transgenic approaches for iPSC-derived skeletal muscle cells”. There are currently no studies on the high-intensity focused ultrasound stimulation of human iPSCs.

Low-intensity pulsed ultrasound is based on frequencies between 40 kHz and 5 MHz, with intensities ranging from 0.02 to 1 W/cm^2^ spatial average temporal average, treatment durations of 5–20 min per day, and duty cycles typically set at 20%^106^. With this regime, ultrasound waves maximize (mild) mechanical effects, minimizing thermal ones.

Low-intensity ultrasonic waves significantly modulate the expression of several genes in human mesenchymal stem cells, regulating cell adhesion, proliferation, differentiation, cytokine, and growth factor production^107^.

In the field of skeletal muscle tissue engineering, Salgarella et al. developed an engineered set-up with high control of the ultrasound dose delivered to the cells (Fig. 3e). On a murine cell model (C2C12 cells), the authors demonstrated that certain frequencies and intensities are more efficient than others in promoting myotube development^108^. They found that stimulation at 1 MHz and 500 mW/cm^2^ was the most effective to achieve high fusion index values and more developed myotubes. Their study highlights the importance of standardization in ultrasonic stimulation experiments. In fact, the lack of appropriate standardization and properly dose-controlled set-ups has negatively affected both in vitro and in vivo studies, thus explaining the contradictory results in the literature concerning the most effective ultrasound stimulation parameters to promote bioeffects^109–112^.

Low-intensity pulsed ultrasound has been used to stimulate human iPSCs, but mainly to boost neural differentiation and to regenerate injured peripheral nerves^113–115^. Although relevant, as yet there have been no reports on using low-intensity pulsed ultrasound to boost the myogenic differentiation of iPSCs.

#### Altered gravity

Gravity plays a vital role in life, from the specification of cell types to the location and size of internal organs, up to the evolution of the species^116^. Altered gravity conditions impact on cells and tissues, including skeletal muscle. Long-term residence in space in microgravity conditions, produces biological adaptations of human skeletal muscle, and muscle loss in particular^117^. Experiments on mice at the International Space Station demonstrated that a key role is played by the E3 ubiquitin ligase MuRF1, which determines the degradation of the contractile apparatus of skeletal muscle^118^. In vitro experiments mimicking such conditions through rotating cell culture systems, highlighted that microgravity did not alter myocyte proliferation, but inhibited cellular differentiation^119^. Recently, the involvement of the PLD2-induced Akt/FOXO regulatory axis was highlighted^120^. Similarly to the results on myocytes, microgravity experiments on iPSCs revealed that lack of gravity preserves greater stemness and inhibits their differentiation^121^. Differentiation of iPSCs in a few phenotypes has been investigated in microgravity, including cardiac phenotypes^121^. However, no studies have been performed on iPSC-derived skeletal muscle cells/tissues.

On the other hand, hypergravity, which can be mimicked through large centrifuges, has a reversal effect on muscles^122^. Experiments on myoblasts revealed that a 2 h exposure at 5 g, 10 g, and 20 g enhanced both cell proliferation and differentiation^123^. Murine ESCs were exposed to parabolic flight-induced acute hypergravity, which led to an upregulation of several genes belonging to developmental processes^124^. However, no studies have been carried out on human iPSCs. Based on the available evidence, hypergravity seems to have a promising role for enhancing the skeletal muscle differentiation of iPSCs.

### Biomaterials for tissue engineering

Skeletal muscle cells grow and differentiate into compact and anisotropic tissue with intimate contact with the various layers of the extracellular matrix (ECM) (see Section “Skeletal muscle tissue embryonic development and architecture”). The chemical interactions between the differentiating myoblasts and the ECM are trivial during developmental processes and in the differentiated tissue, where myotube force transmission is highly dependent on these interactions. Biomaterials that mimic ECM features are crucial for controlling iPSC fate and iPSC-derived skeletal muscle cell functionality.

Biomaterials for skeletal muscle tissue engineering are mainly divided into two groups: (1) natural materials e.g. collagen, fibrin, alginate, Matrigel^®^, hyaluronic acid, gelatin, silk fibroin, chitosan, decellularized tissue ECM; and (2) synthetic materials (e.g. poly(glycolic acid), poly(lactic‐co‐glycolic acid), poly(vinyl alcohol), poly (glycerol sebacate)^33,125^. Natural biomaterials have inherent biocompatibility as well as several biochemical cues fostering cell adhesion and differentiation. Synthetic biomaterials must be functionalized with cues mimicking the biological environment, but their mechanical and structural features can be easily tuned to achieve the desired characteristics.

**Fig. 4:**
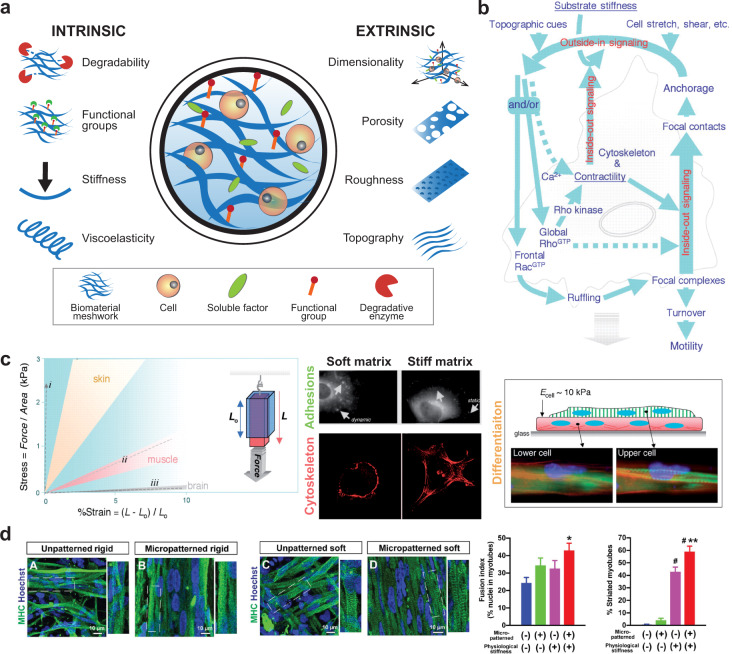
Biomaterial features and their effects on skeletal muscle cells. **a** Scheme of biomaterial properties relevant for cell/tissue engineering, divided into intrinsic and extrinsic ones. **b** Scheme of the intracellular biochemical cascades triggered by the stiffness of the extracellular environment. Images reproduced with permission from^126^. **c** Top: stress/strain curves for different soft tissues (skin, muscle, and brain) from which the slope E can be extracted, representing the Young’s elastic modulus. Dashed lines represent (i) polylactic acid; (ii) artery-derived acellularized matrix; (iii) Matrigel^®^. Bottom: influence of soft and stiff matrix on actin cytoskeleton assembly, cell spreading, and myotube differentiation. Images reproduced with permission from^126^. **d** Left: immunofluorescence staining of iPSC-derived myotubes at two weeks of differentiation on different substrates. Right: evaluation of the fusion index and percentage of striated myotubes in the different conditions. (*N* = 10 fields). *p < .05 versus unpatterned rigid. #*p* < .05 versus unpatterned soft and micropatterned rigid, and **p < .05 versus unpatterned soft. Images adapted and reproduced with permission from^30^.

Choosing the right material with specific tunable features is therefore crucial when designing a supportive myogenic environment (Fig. 4a).

Biomaterial stiffness is key in the interaction between scaffolds and cells. Cells can “feel” the environmental stiffness by pulling against the matrix through focal adhesions^126^ (Fig. 4b, c). The force exerted to deform the matrix influences the response of these mechanotransducers, generating different intracellular signals thus influencing cell fate. The difference between the stiffness of the cells (a few kPa) and the material used for cell culture, such as glass or polystyrene (a few GPa), can impair myotube contractions and typically results in myotube detachment, thus shortening culture duration. Stem cell lineage specification is also strongly influenced by environmental stiffness^34^. Regarding skeletal muscle tissue, MuSCs respond to environmental stiffness by showing a greater self-renewal ability in vitro and in vivo if cultured on soft hydrogels (~12 kPa), compared to more rigid substrates (10^6^ kPa)^127^.

The effects of different substrate stiffnesses (3.5-141 kPa) on the differentiation of MYOD1-reprogrammed iPSCs during early myogenesis have been studied and no influence was found in the skeletal muscle differentiation of iPSCs into iPSC-derived myogenic progenitors concerning this range of stiffness^128^.

However, studies on the terminal differentiation of iPSC-derived progenitors have shown that a soft patterned substrate (85-μm width lanes of Matrigel® patterned on 15-kPa-soft polydimethylsiloxane) can promote later stages of differentiation, such as myoblast fusion and the formation of striated myotubes (Fig. 4d)^30^.

In fact, another crucial factor for skeletal muscle tissue development, is the scaffold anisotropy. Myofibers have a strongly anisotropic organization that maximizes their force transmission (Fig. 2). The anisotropic topography of the substrate strongly influences cell differentiation by addressing skeletal muscle cells in a preferential direction and also driving muscle differentiation^129^. iPSC-derived skeletal muscle cells from healthy donors, as opposed to cells derived from dystrophic patients, align nearly perpendicular to anisotropic nano-grooves. Alteration of the DPC impairs the cell capacity to self-align and form a densely packed SM bundle^130^.

iPSC culture methods in growth conditions typically entail irradiated mouse embryonic fibroblasts (iMEFs) as a feeder layer, and therefore the use of this co-culture for skeletal muscle tissue engineering seemed quite difficult at the beginning. The introduction of feeder-free culture methods has created more defined culture conditions by using recombinant proteins (e.g., vitronectin, laminin) or complex hydrogels (e.g. Matrigel^®^), thus easing the process of keeping the stem cells in an undifferentiated state. These coatings are needed for long-term cultures of iPSCs, and they are sometimes specifically optimized for defined growth media to support iPSC growth and to maintain pluripotency.

The most common coating in iPSC myogenic induction protocols is Matrigel^®^ (see protocols in Table 1 and Table 2), with some protocols using type I collagen^13,21,131^, gelatin^16,59^, or iMEFs^14^. Matrigel^®^ is a natural matrix extracted from Englebreth‐Holm‐Swarm sarcoma in mice. It mainly consists of a mixture of laminin, type IV collagen, entactin, and several growth factors (e.g., FGF2, EGF, IGF-1, TGFβ, PDGF, NGF)^132^. Thanks to its composition, Matrigel^®^ creates a supportive and rich environment for iPSCs, but the variability of the concentration of the ingredients can cause reproducibility problems and tumorigenic response if implanted. In fact, Hughes et al. demonstrated only a ~53% batch‐to‐batch similarity^132^. Nevertheless, also in the creation of iPSC-derived 3D skeletal muscle constructs, Matrigel^®^ has been extensively used to form hydrogels, with the addition of fibrinogen, hyaluronic acid, gelatin, or fibrin^23,46,133,134^.

Fibrin is one of the ideal candidates for natural biomaterials and scaffolding proteins. Fibrin is a protein derived from the action of the protease thrombin on fibrinogen, and it has a strong interaction with the myotube integrins (integrins α7 and α5), thus optimizing the efficiency of force transmission^33^. It is a developmental matrix and compared to other frequently used proteins such as collagen (an adult matrix), it is less stiff and more compliant to muscle cell contraction^135^.

However, transformed cell lines such as C2C12 display a natural fibrinolytic activity as a result of the high levels of plasminogen production. To solve this issue, some anti-proteolytic agents have been used such as aprotinin, genipin^135^, and aminocaproic acid^136^. Maffioletti et al.^21^ resuspended dystrophic iPSCs and healthy controls in a composite of fibrin gel and Matrigel^®^, and showed how they can be differentiated towards a multilineage isogenic culture system with endothelial cells, pericytes, myofibers, and motor neurons spreading from neurospheres placed above the 3D construct. A similar hydrogel composition has also been used to embed differentiating myogenic progenitors, forming aligned myotubes exhibiting electrical responsivity^15^.

Given the hierarchical and modular structure of SMT, 3D bioprinting is becoming increasingly common as it can create macroscopic constructs in a layer-by-layer fashion, by depositing cells and biomaterials simultaneously with a high resolution. The features of the biomaterial used are crucial, and there is an increasing need for smart bioinks supportive for myogenic differentiation and effective for printing^126^.

**Fig. 5:**
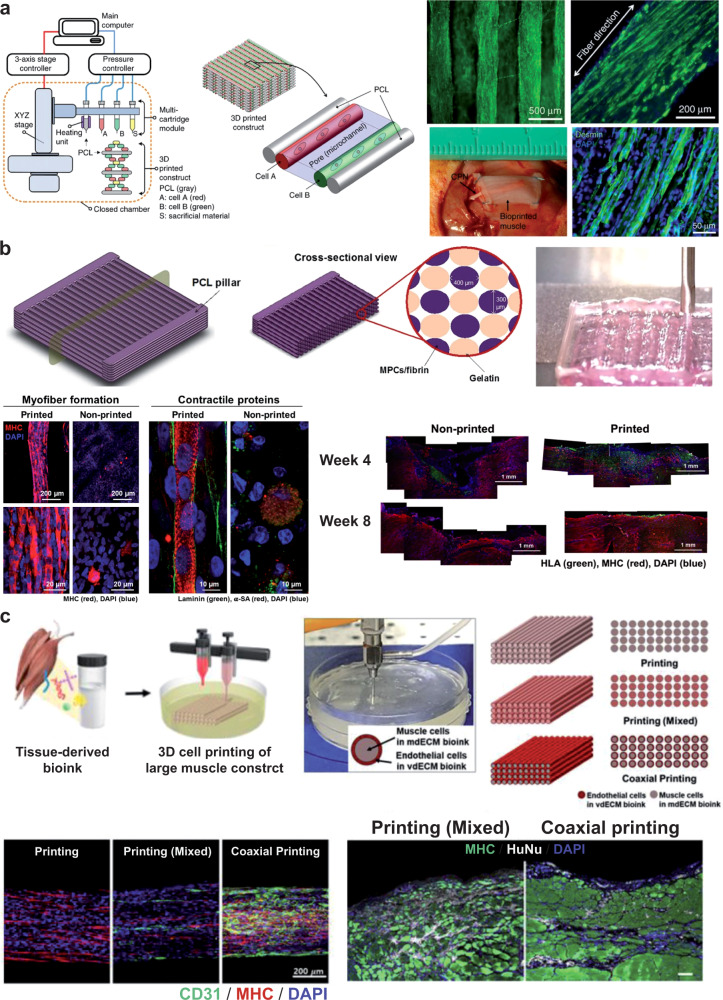
3D bioprinting for skeletal muscle tissue engineering. **a** Left: the ITOP system and its major units, and illustration of the targeted 3D architecture. Right: staining for myosin heavy chain after 7 days of differentiation (top) and image of the construct and desmin staining after in vivo implantation (bottom). Images adapted from^137^. **b** Construct based on a cell-laden bioink made of gelatin, fibrinogen, hyaluronic acid, and glycerol. MPCs muscle progenitor cells. Top: fabrication procedure based on an ITOP system. Bottom left: in vitro results of bioprinted and non-bioprinted (bulk) system. MHC myosin heavy chain. Bottom right: in vivo results after implantation in rat muscle defect models. Images adapted from^133^. **c** Top: construct based on decellularized extracellular matrix-derived bioinks, laden with muscle cells and endothelial cells, organized in different patterns. Bottom left: results of in vitro differentiation, in terms of expression and organization of endothelial marker CD31 and of myosin heavy chain. Bottom right: results of the in vivo experiments. Scale bar: 100 µm. HuNu: human nuclei. Images adapted from^140^.

A milestone in this field is the work of Kang et al*.*^137^. In 2016 developed a bioprinting approach based on an integrated tissue-organ printing (ITOP) system that can generate a 3D freeform shape (Fig. 5a left). The authors fabricated a 15 mm × 5 mm × 1 mm skeletal muscle construct using a bioink composed of fibrinogen, gelatin, hyaluronic acid, and glycerol embedding C2C12 myoblasts and Pluronic F-127 as a sacrificial bioink. After printing, a thrombin solution was used to crosslink the fibrinogen in the cell-laden bioink thereby stabilizing it, while the sacrificial bioink was washed out, to obtain void spaces thus enhancing the diffusion of oxygen and nutrients in the internal regions of the construct. In vivo experiments were also performed by implanting subcutaneously (ectopically) on rat structures differentiated for seven days. The dissected distal end of the proximal stump of the common peroneal nerve was embedded within the construct to promote integration. The results highlighted a well-organized muscle fiber structure (Fig. 5a right), the presence of acetylcholine receptor, nerve integration, and vascularization throughout the muscle constructs indicated by endothelial cell marker expression.

More recently, other bioprinting approaches have been used, for example custom extrusion-based systems, microfluidic printing, inkjet printing, and fused deposition modeling with surface coating. Various bioinks were tested, such as collagen, alginate, polyethylene oxide, polyethylene glycol, silk, and methacrylated gelatin^138,139^.

The ITOP technique and a gelatin fibrinogen-based bioink were proposed again in 2018 by Kim et al*.*^133^., who fabricated a bioengineered SMT based on human primary muscle progenitor cells harvested from biopsies of human gracilis muscles and exploiting the ITOP system (Fig. 5b top). They obtained a macroscopic construct (15 × 15 × 15 mm^3^) with integrated void microchannels (~200 µm wide) between the cell-laden patterns, facilitating the diffusion of oxygen and nutrients. The cells were laden in a bioink composed of fibrinogen, gelatin, hyaluronic acid, and glycerol (similarly to Kang et al.), while the sacrificial bioink (used to generate the void microchannels) was only composed of gelatin, hyaluronic acid, and glycerol. Their construct induced myofiber and contractile protein formation in vitro, with respect to the bulk (non-printed) hydrogel (Fig. 5b bottom left). In vivo results on rats with muscle defects also showed that bioprinted constructs performed much better (Fig. 5b bottom right).

Bioinks can also be based on decellularized ECM. Choi et al. proposed a skeletal muscle and a vascular decellularized ECM bioink, using the porcine tibialis anterior muscle and the descending aorta, respectively^140^. The first bioink was laden with human skeletal myoblasts, and the second one with endothelial cells (HUVEC). These cell-laden bioinks were printed in different configurations, and mixed and organized into a coaxial structure (Fig. 5c top). Choi’s results showed the formation of myotubes in vitro, with the presence of an endothelial network throughout the construct in the coaxially printed structure (Fig. 5c bottom left). In vivo, well-organized *de novo* muscle fibers were found in the coaxial printing, whereas severe scar tissue deposition was observed in the mixed group (Fig. 5c bottom right). These findings highlight the importance of cell co-culture for achieving a good muscle maturation, and that cell spatial organization during fabrication is a key aspect.

Human iPSCs were bioprinted for the first time by Jodat et al*.*^141^, who used a bioink based on gelatin and methacrylated gelatin (which was photocrosslinked by UV light), laden with iPSC-derived muscle progenitor cells. The authors also proposed a non-conventional bioprinting approach, exploiting a pre-gelled methacrylated gelatin supporting matrix as a block, within which the cell-laden bioink was printed by producing vertical lines (instead of the usual horizontal lines). The authors suggested that the role of this methacrylated gelatin supporting matrix is comparable to the hierarchical ECM structure of the native endomysium, a connective tissue that physically supports densely bound aligned myofibers. Jodat used a thermo-reversible gelatin bioink as the sacrificial one to create a perfusable construct, and the 3D pre-vascularized tissue construct was successfully implanted in a volumetric muscle loss-injured animal model. The results were promising and highlight that Jodat’s approach could be used for VML repair.

## Challenges in the clinical translation of iPSC-derived skeletal muscle

In a clinical setting, iPSCs have many advantages over other stem cells, such as MuSCs, ESCs, or mesenchymal stem cells. Although MuSCs in vivo are capable of extensive self-renewal for muscle regeneration in the case of injury or tissue degeneration, only the injection of freshly isolated MuSCs enables robust engraftment and in situ self-renewal^68^. Clinical trials require a considerable number of cells, and MuSC in vitro expansion deeply impairs their regeneration capacity^68^. The problem with ESCs is that they are isolated from the inner mass of a blastocyst with difficult and inefficient protocols, and their use in clinics raises various ethical issues.

On the other hand, mesenchymal stem cells have a high potential as they tend not to stimulate an immunogenic response. However, the invasive process needed for their isolation and the myogenic potential of less than 40% means that much progress is needed before they can be used in a clinical setting^142^.

iPSCs can be extensively expanded in vitro, leading to a high number of cells to be transplanted or differentiated potentially towards any phenotype. No external donors are needed since isogenic iPSCs can be generated from different sources, such as fibroblasts from a non-invasive cutaneous biopsy or T cells from peripheral blood. Furthermore, gene-editing techniques such as the CRISPR/Cas9 system can be used to correct molecular defects.

There are however limitations in using iPSCs for clinical trials. The iPSC genesis and the reprogramming processes of somatic cells are characterized by epigenetic remodeling and alterations in the chromatin structure. These alterations and changes may modify the iPSC phenotype, while in other cases the residual epigenetic memory from the somatic donor cell source may reduce the pluripotency of the generated cell line, leading to a biased differentiation potential^143^.

Several reviews have highlighted that the reprogramming process can be very inefficient (with a yield of less than 1%^144^), and the cost of generating a patient-specific clinical-grade iPSC cell line is high, around U.S. $800,000^145^. Reprogramming methods range from using integrative viruses, such as retroviruses and lentiviruses, to non-integrative technologies with adenoviruses, PiggyBac transposons, Sendai viruses, episomal vectors, or recombinant proteins. These non-integrative technologies avoid possible insertional mutagenesis, though some genome alterations can be inherited from the somatic donor cells. Genomic alterations can cause aberrant phenotypes after implantation with possible teratoma development, and although preclinical studies have not noted signs of teratoma formation, this concern remains.

Isogenic iPSCs are widely known to be immune-privileged, and one study on the immunotolerance of undifferentiated iPSCs and iPSCs derivates demonstrated that only iPSC differentiation led to a tolerogenic immune response^146^. Other studies showed no lymphocyte or macrophage infiltration after transplantation of iPSC-derived dopamine neurons into primate brains over two years, without any immunosuppression^147^. Allogenic iPSCs with different human leukocyte antigens could be exploited in a universal transplantation technology^148^.

Due to the high variability of iPSC lines (e.g., batch-to-batch, clone-to-clone), the huge cost, and possible immune rejection, iPSC banking services have been established in the last ten years. They aim to standardize iPSC culture and handling, according to good manufacturing practices and quality standards^145^. Although these services can reduce the variability at the beginning of the iPSC genesis and handling procedure, subsequent steps can also be taken, for example, (clone selection, differentiation protocols, reagent lots, and experimental conditions. Other standardized controls must therefore be introduced in the following phases, including the absence of mycoplasma contamination, checking for normal karyotypes, no chromosomal aberrations, and no presence of reprogramming transgenes or vectors^149^.

Because of the obstacles encountered in the development of iPSC-based therapies, iPSCs have not been used much in clinics. Deinsberger et al. performed a systematic worldwide analysis of clinical trials involving PSCs, divided into interventional trials, with cell transplants in patients, and observational ones, regarding the generation of patient-specific cell lines used for in vitro testing^150^. Out of a total of 131 clinical trials, 77% were observational and only 23% were interventional. Clinical trials involving iPSCs are mainly observational (only 27% of the interventional studies involve iPSCs), meaning that a solid basis for the clinical translation is under development, but many challenges remain.

With regard to skeletal muscle tissue engineering, there have been no interventional clinical trials using iPSC-derived muscle cells. To date, patient-derived iPSCs have mainly been used such as in vitro tools to model muscular diseases, to study the pathological molecular mechanisms, and for drug testing before in vivo translation^151^.

The use of gene-editing technologies can be valuable by providing isogenic healthy control cell lines. Some preclinical studies have implanted iPSC-derived myogenic progenitor in animal disease models, as described in Table 1 and Table 2. There are no effective treatments for these genetic conditions, with clinical trials aimed at correcting the molecular defect in order to restore protein expression by gene replacement strategies with RNA-based (conventional or based on the CRISPR/Cas9 system) or cell-based approaches. Interestingly, a study on the transplantation of iPSC-derived mesangioblast-like cells expressing the α-sarcoglycan (SGCA) gene into limb-girdle muscular dystrophy 2D mice (*Sgca*^null^) succeeded in generating SGCA+ muscle fibers^12^. The autotransplantation of genetically-corrected iPSCs is promising, however the main issues are the poor survival and migration of the iPSC-derived progenitor cells. The tissue atrophy and degeneration caused by the abovementioned muscular disorders are associated with an inflammatory environment and the loss of tissue integrity, which considerably impair cell viability and migration^152^.

On the other hand, for conditions such as VML, with the need to substitute most of the muscle, the clinical approach must focus on the fabrication of meso/macroscopic 3D constructs, recapitulating the skeletal muscle architecture complexity and the representation of the resident cell populations. This is necessary both for the prodifferentiative effect that these populations have on skeletal muscle cells (e.g. fibroblasts, motor neurons), and the need to fabricate terminally differentiated functional tissue.

As described in Section “Skeletal muscle tissue embryonic development and architecture”, several cell populations are found in skeletal muscle tissue, including muscle-resident populations such as cells from the vasculature, fibroblasts from the connective tissue wrapping the muscle, tenocytes, adipocytes, fibro-adipogenic progenitors, and motor neurons. In vitro fabrication of a construct recapitulating a functional skeletal muscle tissue therefore involves several steps. Most of the muscle-resident cells are in the G0/G1 state, highlighting the low turnover of the skeletal muscle tissue^153^. Knowing the development path and the morphogenetic signaling of the individual phenotypes is crucial to better engineer the co-culture of different cell populations, thus to lead a concert of all the different differentiation timings, morphogens, and growth factors.

Future evolutions of 3D bioprinting will open up interesting scenarios in this domain. Fabrication techniques are evolving to produce macro-structures that are as packed as possible, stable over time, and with a micrometric resolution. Recent interesting examples of new bioprinting approaches include continuous chaotic bioprinting, which led to the fabrication of hierarchically-structured engineered muscle-like constructs in a continuous and simple fashion^154^, fibrillation/leaching of poly(vinyl alcohol) (included in the bioink) after the printing process, to create a uniaxially aligned micro-topographical structure in the printed construct, thus promoting self-alignment of muscle cells^155^ and electric-field assisted bioprinting for aligning a cell-laden bioink^156^ or to promote myogenesis^157^.

There are several interesting innovations regarding bioinks, and newly formulated molecules and polymers may be an exciting route. However, traditional polymeric formulations are also promising if they are supplied with active nanomaterials that can turn these formulations into multifunctional ones^112,158^. These evolutions, together with the use of iPSCs in the printing process, will advance the field considerably.

The combination of chemical, topographical, and stiffness-related cues on the one hand (ensured by appropriate biomaterials and ad hoc fabrication technologies) and biophysical stimuli (provided through mechanical, electromagnetic, and other types of energy transfer to cells), has barely been explored in the state of the art for iPSCs. However, all these approaches have the potential to recapitulate part of the intricated series of dynamic inputs that the cells receive during embryogenesis.

Several questions still need to be answered: (1) What is the optimal modality and energy dose for each stimulus, to drive the expression of a certain phenotype, at a given time-point? (2) When and how should different stimuli be combined to enhance this effect, and which ones should be used? (3) What is the weight of each stimulus used for this purpose, depending on the desired target tissue type, and how does this weight vary over time?

Answering these questions would considerably advance the field of skeletal muscle tissue engineering, as well as other regenerative medicine domains. To pursue this objective, systematic experiments should be designed, exploring the “sequence space” of the various possible combinations of the above-mentioned stimuli over time thereby creating a clear map of the cell response. However relying only on experiments, this is an enormous task. Mathematical modeling and advanced data processing techniques (e.g. artificial intelligence, and in particular deep learning) may make a difference^159–162^, by reducing the complexity of this task and predicting cell response patterns more reliably.

In addition to bioengineering platforms to enhance iPSC differentiation, it is also worth considering platforms that monitor the performance of these constructs. For example, a contractile force screening system can be created using custom multiwell plates with microcantilevers^163^.

Active prostheses control is an alternative field of skeletal muscle tissue engineering. Such control is generally achieved by superficial electromyographic sensors placed in the socket of the prosthetic device and in contact with the residual limb. The control of a single degree of freedom per time is carried out by applying a simple threshold or a proportional amplitude method on superficial electromyographic sensor signals recorded from antagonistic muscles (e.g., wrist flexor and wrist extensor). In the case of prosthetic devices endowed with several degrees of freedom, but still with two control signals, switching among degrees of freedom is normally achieved by co-contraction, as in finite state machines. This serial operation is slow and unnatural; in addition, it requires considerable training and cognitive effort in the execution of the task^164^.

Multi-fingered prosthetic hand control techniques based on machine learning and targeted muscled re-innervation^165^ are interesting alternatives but they are still constrained by a few independent control signals.

The regenerative peripheral nerve interface technique re-innervates a muscle portion removed from another body region, preventing the need for denervation^166^. However, regenerative peripheral nerve interfaces clearly entail, albeit limited, further harm to the patient.

Consequently, an engineered in vitro muscle, as well as multifunctional afferent and efferent artificial structures, are of great technical and scientific interest to overcome the drawbacks and to maximize the performance of the afore-mentioned prosthetic control techniques. Such an improvement in the control would promote greater functionality of the prosthetic device, with clear clinical benefits for the amputees. Human iPSCs hold great promise for this application, at the frontier between robotics and bioengineering.

### Co-culture of skeletal muscle cells with muscle-resident phenotypes

#### Vascular cells

The cellular components of the vascular network (endothelial cells, vascular smooth muscle cells, and pericytes) share a different embryonic origin depending on the localization of the anatomical location of the vasculature^167,168^. Endothelial cells, which line the vessel lumen, start the process.

**Fig. 6:**
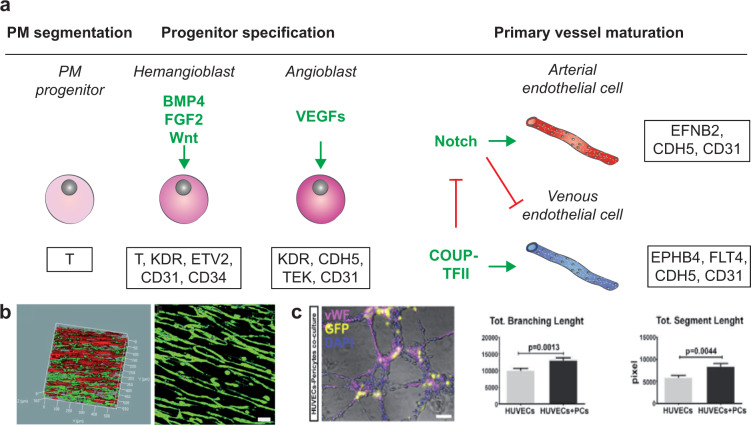
Vasculogenesis, endothelial cell development, and co-culture with skeletal muscle cells: influence on myogenesis. **a** Representation of the formation of primary vessels during vasculogenesis, with endothelial cell differentiation starting from the paraxial mesoderm (PM) progenitor. Marker genes are shown in the bottom boxes, while the main signaling molecules are indicated in green if acting as pro-differentiative actors, in red if they inhibit the differentiation process. **b** Fluorescence confocal images of a co-culture of HUVECs (50%, in green) and muscle cells (50%, in red) in a fibrin matrix (left image). Focus on the endothelial network formation of HUVEC alone (right image). Scale bars: 50 μm. Images adapted and reproduced with permission from^177^. **c** Fluorescence image of HUVECs (von Willebrand factor, magenta) in co-culture with primary pericytes (GFP) showing the formation of a network. Nuclei were identified by DAPI staining (blue). The graphs show the quantification of the tubular structures, in terms of total segment length, total mesh area, and total branching length of HUVECs without (grey columns) or with pericytes (black columns). Images adapted and reproduced with permission from^19^.

A combination of BMP, FGF2, and WNT signals activate the transcription factor ETV2 in the lateral plate mesoderm leading to the formation of endothelial cell precursors, the hemangioblasts, from the splanchnic mesoderm (Fig. 6a).

Subsequently, the expression of FLT1 (VEGFR-1), KDR (VEGFR-2/Flk-1), and the receptors for the vascular endothelial growth factors A (VEGF-A) on the hemangioblast membrane start the differentiation and proliferation into angioblasts. Under VEGF-A guidance, cells begin sprouting new vessels and eventually undergo arteriovenous specification^168^. In the subsequent angiogenesis, vessel maturation occurs, with the recruitment of mural cells (pericytes and vascular smooth muscle cells) and the expansion of the vessel network. Through the tyrosine-kinase transmembrane receptors transcribed from the TEK gene (TIE), angiopoietins mediate the interaction between endothelial cells and pericytes, which are recruited by the endothelial cells^169^.

More than other cell types, vascular smooth muscle cells have multiple embryonic origins, as reviewed by Majesky^170^. However, the majority of smooth vascular muscle cells generally have a mesodermal origin, specifically from somites of the ventral posterior sclerotome^170,171^.

Although pericytes express canonical markers of mesenchymal stem cells such as CD29, CD44, CD73, CD90, CD105^172^, and are negative for CD31, PAX7, and MYOD1, they are very heterogeneous in terms of marker expression origin, and morphology^44,173^. The chondroitin sulfate proteoglycan 4 (*Cspg4* or *Ng2*) has been used as a pericyte marker in murine models during vascular morphogenesis^174^. Pericytes can differentiate towards adipogenic, chondrogenic, osteogenic, and myogenic lineages, however the mechanisms regulating pericyte myogenic differentiation are still mostly unknown. There are many hypothesesregarding their embryological origin, and the literature suggests that they most probably share the same lineages as vascular smooth muscle cells, depending on the organ studied^44^.

The capillaries found inside the skeletal muscle tissue are exclusively composed of endothelial cells and pericytes, and a lack of vascularization limits the size of the engineered tissue because of the limited diffusion of nutrients and oxygen. One of the options is therefore to pursue scaffold vascularization through in vivo angiogenesis, by implanting a 3D scaffold and developing techniques to stimulate host vessel ingrowth. This can be achieved by adding VEGF in the scaffold or by anastomosing the construct to the host vasculature^175^, although the infiltrating vessels often remain limited to the construct periphery. Another option is the pre-vascularization of the scaffold in vitro through vasculogenesis, thus entailing a co-culture of myoblast and endothelial cells. Rosa et al*.* were able to generate functional arterial and venous-like endothelial cells from human iPSCs^176^. The co-culture option involves the fabrication of an a priori perfusable construct, thus easing vascularization and survival upon implantation. Skeletal muscle and endothelial cell co-culture systems can be based on 3D microporous scaffolds recreating the skeletal muscle tissue architecture, or 3D bioprinted constructs embedding vessel-like structures. Gholobova et al*.*^177^ established a co-culture of a mixed population of primary muscle cells (with 8% fibroblasts) and HUVEC in a compact 3D fibrin gel. Seven days of differentiation led to an advanced vascular network, without hampering myoblast fusion and differentiation, thanks also to the proangiogenic activity of the fibrin gel (Fig. 6b). However, the primary muscle cells did not show a high level of myogenic differentiation, with the myotubes not showing the canonical sarcomere striated ultrastructure, thus leaving margins for further improvements. Nevertheless, the presence of endothelial cells alone may not be sufficient to create a spread vascular network. Pericytes during HUVEC vasculogenesis have in fact been demonstrated to foster endothelial cell branching and the total length of the vessels compared to HUVECs alone^19^ (Fig. 6c).

#### Fibroblasts

The connective tissue cellular components are the fibroblasts. Different methods have been proposed to identify them, e.g., by staining collagen, the intermediate filament vimentin, or through localization in the interstitial ECM. These features cannot be defined as fibroblast-specific markers or features since, for example, the myocytes themselves can secrete ECM proteins^178^. The great heterogeneity of fibroblasts highly depends on their activity and their localization. Being tightly associated with the skeletal muscle tissue, fibroblasts share with it most of the embryonic sites of origin. In terms of skeletal muscle development, appropriately coordinated development of muscle and connective tissue lineages (also including tendons, which will be described later) is required for the formation of the musculoskeletal system^179^. There are various possible fibroblast origins, from differentiation from hematopoietic stem cells, pericytes, monocyte subpopulations, to epithelial-mesenchymal transition from epithelial cells^180^.

Intramuscular connective tissue fibroblasts throughout the body express the platelet-derived growth factor receptor alpha (PDGFRA), the transcription factor TCF4 (TCF7L2), and the T-box transcription factor 5 (TBX5), while a limb subpopulation expresses the odd-skipped related transcription factor 1 (OSR1)^181^. Due to the overlapping of some molecular markers and similar roles during muscle homeostasis, fibroblasts from intramuscular connective tissue and bipotent fibro-adipogenic progenitors may be identified as the same cell population, as recently suggested by Sefton and Kardon^182^. Fibro-adipogenic progenitors (PDGFRA+/ATAXIN1+/CD34+) are mesenchymal cells, developmentally distinct from myogenic progenitors, and highly abundant in the adult muscle^153^. They are a source of fibroblasts and adipocytes during muscle injury scarring and fibrosis. In the quiescent state, they are localized near the blood vessels outside the capillary basement membrane^183^. Fibro-adipogenic progenitors maintain the MuSC pool^184^, and secrete pro-myogenic factors (IL-6, WNT, IGF-1), increasing the terminal differentiation of myogenic progenitors^185^. The role of fibroblasts in myogenesis has been largely neglected, mainly focusing on their actions during fibrosis and muscle regeneration. Recent studies have instead highlighted that fibroblasts influence muscle development, morphogenesis, and localization, as reviewed in^182,186^. As the muscle develops, the intramuscular connective tissue surrounds the epaxial and hypaxial muscles, with the intramuscular connective tissue fibroblasts intercalated amongst the myogenic cells in the myotome. Thanks to the easier accessibility in avian and murine models, a great number of studies have been carried out on the role of intramuscular connective tissue fibroblasts on the development of limb muscles. Somitic PAX3+ myogenic precursor cells migrate towards the limb bud, and fibroblasts regulate muscular patterning by the chemoattractive action of CXCL12 chemokine on CXCR4+ muscle progenitors, and the chemorepulsive role of EPHRINA5 on EPHA4+ muscle progenitors^186^. TCF4 gain- and loss-of-function experiments have been performed on murine models, and a muscle mispatterning occurred^186^.

Over the years, different protocols have been developed to isolate fibroblast-free MuSCs, aimed at obtaining a pure myogenic culture in which fibroblast proliferation would not take over on the low-proliferative MuSCs. Other groups on the other hand, exploited this intrinsic co-culture and demonstrated the possibility of exploiting fibroblasts for the fabrication of 3D rolled skeletal muscle constructs, such as the pioneering work of Dennis et al*.*^187^. The role as a support in the skeletal muscle cell auto-assembly observed during embryonic development has been confirmed.

**Fig. 7:**
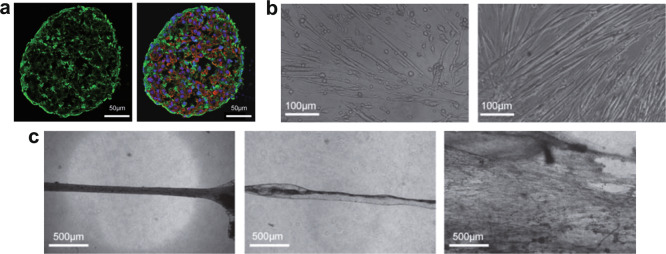
Fibroblast co-culture with skeletal muscle cells: influence on myogenesis. **a** Fluorescence images of enhanced-GFP-expressing mouse embryonic fibroblasts (MEFs) evenly distributed in a co-culture with primary mouse myoblasts (PMM), stained for fast myosin heavy chain (red) and nuclei (blue). **b** 2D myotube monocultures degenerated after 18 days in culture (left), while the presence of fibroblasts in co-culture drastically enhanced their stability (right). **c** Left: MEF/PMM co-culture led to the assembly into a 3D fibrin construct with consequent fibrin degradation. MEF monoculture assembled in a 3D construct (middle), but PMM monoculture without MEFs did not show any 3D autoassembled construct or fibrin degradation (right). Images adapted and reproduced with permission from^188^.

In a culture of mouse embryonic fibroblasts and MuSCs at a ratio of 1:1, fibroblasts were distributed throughout the tight structure without impairing the SM contractile forces (Fig. 7a) and only fibroblasts and MuSC co-culture showed the formation of a rolled cylindrical structure compared to a monoculture of MuSCs (Fig. 7b). Fibroblasts also contributed to a MuSC higher survival rate and the formation of more hypertrophic myotubes (Fig. 7c)^188^, highlighting the stimulatory effect of fibroblasts on myogenic differentiation and myoblast fusion^189^. In addition, a triculture of MuSCs, HUVEC, and human skeletal muscle fibroblasts evidenced their angiogenic effect by secreting high levels of HGF and promoting VEGF production in MuSCs^190^.

Due to the regulatory role of fibroblasts both in muscle regeneration and in fostering myogenic differentiation, it is clear that fibroblasts are necessary to construct a functional 3D muscle construct in vitro. However, to date no attempts on the co-culture of fibroblasts and iPSC-derived skeletal muscle cells have been performed.

#### Tenocytes

**Fig. 8:**
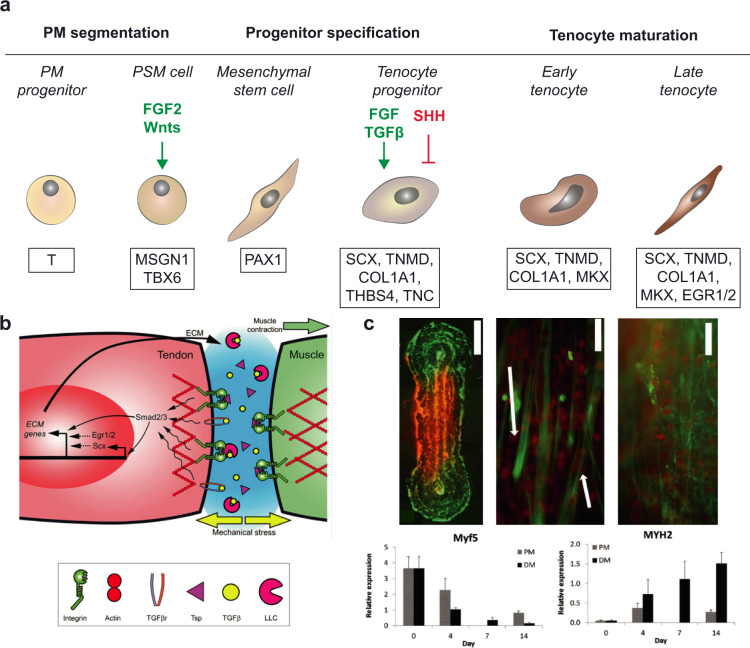
Tenocyte development and co-culture with skeletal muscle cells: influence on myogenesis. **a** Representation of the differentiation process of tenocytes starting from the paraxial mesoderm (PM) progenitors. Marker genes are shown in the bottom boxes, while the main signaling molecules are indicated in green if acting as pro-differentiative actors, in red if they inhibit the differentiation process. PSM presomitic mesoderm. **b** Scheme of the myotendinous junction formation. Image adapted and reproduced with permission from^196^. **c** Top left: 3D printed co-culture of myoblasts (red) and tenocytes (green) just after printing. Scale bar: 2 mm. Top middle: co-culture differentiated for seven days and stained for myosin heavy chain (green) and nuclei (red). Arrows indicate striated and multinucleated myofibers. Top right: focus on the tenocytes in the co-culture stained for type I collagen. Scale bar: 50 µm. Bottom: gene analysis expression of muscle and tendon monoculture in proliferation medium (PM = gray bars) or differentiation medium (DM = black bars). Relative expression is shown as mean ± standard error of the mean (SEM). Images adapted and reproduced with permission from^198^.

Tenocytes are other cellular components of the muscular connective tissue. These are interstitial cells that are also found in adult skeletal muscle, expressing tendon markers such as tenomodulin (TNMD), thrombospondin 4 (THBS4), early growth response 1 (EGR1), collagen type I alpha 1 chain (COL1A1), and scleraxis (SCX), a transcription factor essential for tendon differentiation during development^153,191^ (Fig. 8a). Tenocytes share the same developmental pathway as myogenic cells until the paraxial mesoderm induction^192^. They derive from PAX1+ mesenchymal cells of the sclerotome, generated by the combined action of notochord-derived paracrine factors, especially sonic hedgehog (SHH) (Fig. 8b).

At this point, a clear division of the cell fate from myogenic cells is marked by the expression of muscle regulatory factor inhibitors in the tenocyte progenitors^153,193^. The subsequent induction by FGF, directly secreted by the myotome, and transforming growth factor-beta (TGFβ), leads to the development of the tenocyte progenitors SCX+.

The connection between the future myofibers and tenocytes starts here, with the association of tenocytes and myogenic cells^179^.

Connective tissue plays a role in muscle patterning regulation n *Drosophila melanogaster*, where tendons are involved in muscle patterning and the formation of the myotendinous junction^194^. However, the influence of tendons on muscle tissue development is less clear in vertebrates. In *Scx* mutant mice, the forelimb muscle is impaired, underlying tendon implications only in the late events of muscle morphogenesis^195^.

Tenocytes also play a role in skeletal muscle development through chemical and mechanical signaling between the two tissues in morphogenesis and differentiation. The developmental paths of tendons and skeletal muscle tissue are very closely linked, with the mechanical forces generated by the developing muscle inducing a modification in the ECM composition at the interface between the two tissues under maturation (Fig. 8b). These changes are driven by the action of different mechanoresponsive receptors exposed on the cells, including integrins, integrating outside-in and inside-out signaling. ECM remodeling and changes in mechanical properties induce tenocyte terminal prodifferentiative signals (mechanisms reviewed in ref. ^196^). The strong physical interaction of skeletal muscle cells and tenocytes leads to the formation of the specialized myotendinous junction, whose mechanical integrity and structural characteristic is still difficult to reproduce in vitro. Initial studies have tried to recreate the interface between the two tissues using neonatal or adult explanted tendons in co-culture with primary rat myoblasts^197^. The interface can withstand a tangent modulus of 37.2 kPa ± 10.3 kPa before breaking, but was still poorly organized compared to the highly interdigitated natural structure.

Without exploiting explanted tissue, which can be very difficult to translate in a clinical setting, Laternser et al. used 3D bioprinting for the specific spatial localization of tenocytes and SM cells^198^. They succeeded in the fabrication process using two bioinks (a gelatine methacryloyl-polyethylenglycol dimeth-acrylate-based ink and a pure gelatine methacryloyl based) around two rigid anchor posts, and in the differentiation of the two cell types in co-culture. However, the poor organization of the interface was too weak to withstand the developing tension of the skeletal muscle tissue (Fig. 8c).

#### Motor neurons

Motor neurons derive from a different germ layer, namely the ectodermal region. After ectodermal invagination and the formation of the neural tube and the external ectoderm, the dorsoventral patterning of the neural tube begins thanks to the action of WNT; TGFβ family proteins, and retinoic acid from surrounding regions (Fig. 1b).

**Fig. 9:**
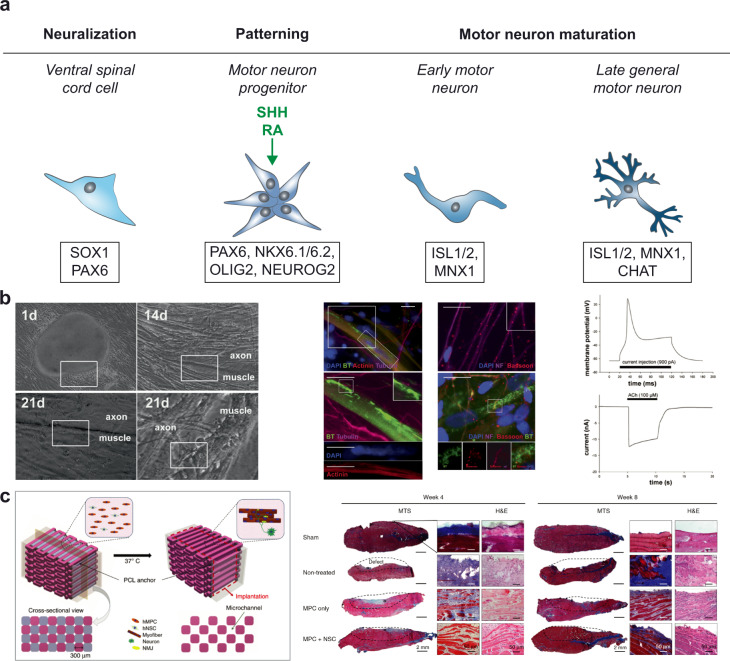
Motor neuron development and co-culture with skeletal muscle cells: influence on myogenesis. **a** Representation of the formation of motor neurons. Marker genes are shown in the bottom boxes, while the main signaling molecules are indicated in green if acting as pro-differentiative factors. **b** Left: representative phase-contrast images of a human iPSC-derived moto-neurosphere at different time points after plating. Small neurites were outgrowing from the moto-neurosphere. In the inserts are shown contacts between neurites and myotubes. Middle left: maturation of acetylcholine receptor clusters and neuromuscular junction formation. Co-culture between human iPSC-derived motor neurons with CD34-enrichment derived myotubes. α-Bungarotoxin (α-BT) labeling after 21 days indicates the formation of a mature neuromuscular junction. Middle right: Bassoon labeling after 21 days indicates presynaptic terminals along the axons (top figure). At the end plate region, a close apposition between presynaptic and postsynaptic markers could be detected, as shown by Bassoon and α-BT stainings (bottom figure). Scale bar: 20 μm. Right: electrophysiological properties of myotubes in culture after differentiation and representative traces of current-clamp measurements and the generation of an action potential following acetylcholine treatment. MP resting membrane potential, AP action potential. Images reproduced with permission from^202^. **c** Left: scheme of a bioprinted construct with the cell-laden bioink, the acellular sacrificing bioink, and the supporting polycaprolactone pillar. Right: histological examination of skeletal muscle regeneration through the 3D bioprinted constructs at 4 and 8 weeks after implantation. Dashed lines: defected area; MTS: Masson’s trichome staining; H&E: hematoxylin and eosin. Images reproduced with permission from^134^.

The ventrodorsal gradient of SHH is crucial: the cells exposed to the highest levels of SHH are the motor neuron progenitors, expressing NK6 homeobox 1 (NKX6.1), the oligodendrocyte transcription factor 2 (OLIG2), and PAX6. The differentiation program starts at this point, stimulated by retinoic acid signaling and OLIG2. OLIG2 together with one of its targets, neurogenin 2 (NEUROG2), contribute to promoting motor neuron fate, by inducing the expression of motor neuron and pancreas homeobox 1 (MNX1 or HB9) (Fig. 9a).

MNX1, which is a specific marker for postmitotic spinal motor neurons, activates its own expression, making the motor neurons independent from SHH and retinoic acid signaling. There are several motor neuron subtypes, which differ in terms of the fibers they innervate, and they follow different specific molecular patterning, as extensively reviewed by Stifani^45^.

Besides being an essential interface for the control of skeletal muscle contraction, motor neurons are a key to terminal myogenic differentiation. In vivo, myotube responsiveness to motor neuron stimulation grows during tissue maturation by increasing the expression of the acetylcholine receptor. The expression of these transmembrane proteins also occurs in vitro, but only motor neurons in co-culture lead to an improved myofiber maturation^35^.

Some studies have aimed to establish a co-culture with iPSC-derived motor neurons and muscle cells,, with different approaches for the challenging formation of neuromuscular junctions^199–201^. Demestre et al. differentiated skeletal muscle cells and motor neurons from isogenic iPSCs in two parallel protocols. They used EB-based methods and enriched the skeletal muscle population for CD34^202^. After the skeletal muscle cells showed contractile properties and responsiveness to acetylcholine, iPSC-derived lower motor neurons were seeded on top of iPSC-derived skeletal muscle cells for 3–4 weeks. Mature neuromuscular junctions were identified at later stages of the co-culture (Fig. 9b).

One protocol with a co-differentiation in a co-culture was developed and pursued for a period of approximately 30 days^203^. Among other differentiation factors, the use of LDN193189, a BMP pathway inhibitor, induced both muscle and neural differentiation. Interestingly, the temporary presence of DAPT, a Notch pathway inhibitor inducing postmitotic motor neuron differentiation, caused the early onset of myotube contractile properties (from two months post differentiation to 19-20 days from the onset of differentiation).

The integration of motor neurons in skeletal muscle cell cultures has not only been performed in 2D dishes, but also in a 3D bioprinted millimeter-scale construct^134^ (Fig. 9c). The construct implantation in a model of extensive muscle defect injury demonstrated its potential for the functional and structural restoration of the damaged muscle. A full restoration of muscle force was observed after eight weeks compared to the sham control, and the skeletal muscle cells aided tissue regeneration by differentiating and forming an organized architecture.

Moving towards a multilineage with four different phenotypes, Maffioletti and coworkers created an iPSC-derived 3D artificial skeletal muscle construct using isogenic iPSCs. They increased the histological complexity of the skeletal muscle construct by adding 30% of iPSC-derived vascular cells (endothelial cells and pericytes) and fibrin neurospheres with neural progenitor cells on top of the 3D culture. This first attempt at integrating such a variety of different phenotypes derived from iPSCs, further confirmed that the maturation of a functional artificial skeletal muscle tissue would strongly benefit from other cell lineages.

## Conclusions

Significant results are possible in skeletal muscle tissue engineering thanks to the use of iPSCs, however various challenges still need to be addressed. Although biochemical differentiation protocols (both transgenic and non-transgenic) have become more efficient, more robust protocols are needed to reduce the output variability when starting with different cell lines. In this view, Van der Wal et al. developed and optimized a differentiation protocol on iPSCs from 15 different donors and performed over 50 individual differentiation experiments, highlighting the importance of protocol reproducibility^18^. Comparing the differentiation efficiency between protocols is still a major problem, mainly due to differences in the reporting methods. High levels of differentiation, for example, can be obtained from the pooled percentage of cells expressing PAX7 or MYOD1, thus preventing a comparison with protocols that do not follow this procedure. Several groups are therefore debating how to establish standards for the evaluation of muscle cell maturation and consequent protocol differentiation efficiency^8,64^.

Future progress in iPSC technology will exploit knowledge from different fields, from embryology to material science and mechatronics for biophysical stimulation.

This review has provided an overview of the engineering tools that can be applied in vitro (in synergy with biochemical protocols) for the fabrication and biophysical stimulation of functional muscle tissues. Such tools include electromagnetic stimulation systems, platforms for providing different kinds of mechanical stimuli, biomaterials, and microfabrication techniques.

The importance of co-cultures has also been highlighted. This 360-degree approach may widen the scope for future applications of iPSC technology in a clinical setting. The challenges ahead include the reprogramming processes leading to a biased differentiation potential, the high variability of the iPSC lines, the hard-to-modify pathological genetic conditions, and difficulties in recapitulating the whole skeletal muscle architecture complexity and a realistic representation of the resident cell populations.

Once these issues have been solved, skeletal muscle tissue engineering may become a viable solution for volumetric muscle loss, but also for other intriguing applications, such as the development of patient-specific muscle-on-a-chip platforms for drug screening and the implementation of novel strategies for the control of active prostheses.

### Note added to proof

Gene and protein names and symbols follow 1) for human: HUGO Gene Nomenclature Committee (European Bioinformatics Institute), available at http://www.genenames.org; 2) for mouse: Mouse Genome Informatics Web Site, available at http://www.informatics.jax.org/.

## Data Availability

The authors declare that the data supporting the findings of this study are available within the paper.
